# Factors other than hTau overexpression that contribute to tauopathy-like phenotype in rTg4510 mice

**DOI:** 10.1038/s41467-019-10428-1

**Published:** 2019-06-06

**Authors:** Julia Gamache, Kellie Benzow, Colleen Forster, Lisa Kemper, Chris Hlynialuk, Eva Furrow, Karen H. Ashe, Michael D. Koob

**Affiliations:** 1 Department of Neurology, N. Bud Grossman Center for Memory Research and Care, Minneapolis, MN USA; 2 Department of Laboratory Medicine and Pathology, Minneapolis, MN USA; 3 BLS Histology and IHC Laboratory, Minneapolis, MN USA; 4 Veterinary Clinical Sciences (College of Veterinary Medicine), University of Minnesota, Minneapolis, MN 55455 USA

**Keywords:** Animal disease models, Alzheimer's disease

## Abstract

The tauopathy-like phenotype observed in the rTg4510 mouse line, in which human tau_P301L_ expression specifically within the forebrain can be temporally controlled, has largely been attributed to high overexpression of mutant human tau in the forebrain region. Unexpectedly, we found that in a different mouse line with a targeted-insertion of the same transgene driven by the same tetracycline-TransActivator (tTA) allele, but with even higher overexpression of tauP301L than rTg4510, atrophy and tau histopathology are delayed, and a different behavioral profile is observed. This suggests that it is not overexpression of mutant human tau alone that contributes to the phenotype in rTg4510 mice. Furthermore we show that the tauopathy-like phenotype seen in rTg4510 requires a ~70-copy tau-transgene insertion in a 244 kb deletion in *Fgf14*, a ~7-copy tTA-transgene insertion in a 508 kb deletion that disrupts another five genes, in addition to high transgene overexpression. We propose that these additional effects need to be accounted for in any studies using rTg4510.

## Abstract (web summary)

The rTg4510 mosue line has a tauopathy-like phenotype which is attributed to overexpression of human tau in the frontal cortex. Here the authors identify potential confounding genetic factors that could contribute to the phenotype.

## Introduction

The widely used rTg4510 mouse model of tauopathy recapitulates key features of these human diseases, including progressive age-related neurofibrillary tangles (NFTs), memory impairment and a dramatic loss of neurons in young mice^1,2^. The rapid onset and severity of neuron loss in this model was particularly surprising when first reported given that earlier mouse models incorporating similar human *microtubule-associated protein tau* (*MAPT*) transgenes with the pathogenic P301L mutation do not develop overt atrophy^3,4^. Unlike these other tau_P301L_ overexpression models, however, rTg4510 uses a responder-driver system to activate transgene expression specifically in forebrain neurons. Responder Tg4510 mice are crossed to a driver line^5^ that harbors a tetracycline transactivator (tTA) transgene to generate bi-transgenic rTg4510 (“r” refers to regulatable) progeny. The particularly high level of tau_P301L_ overexpression specifically in the forebrain of these mice has generally been accepted as the direct cause of the premature gross forebrain atrophy and other tauopathy-like phenotypes in this line, a hypothesis supported by the fact that suppression of tau_P301L_ expression by doxycycline (DOX) halted neuronal loss and improved memory function^2^. Mice from a control line in which wild-type (WT) human tau is expressed at levels roughly equivalent to tau_P301L_ in rTg4510 mice do not develop either progressive memory deficits or overt atrophy, supporting the idea that the tau_P301L_ mutant form of tau in particular was the direct cause of these phenotypes in rTg4510^6^.

Our overall goal when we began the project described here was to determine the precise molecular features of the tau_P301L_ protein that cause the rapid neurodegeneration phenotype characteristic of rTg4510, but we realized that this line was an inadequate starting point for this work. Although the transgene construct used to generate Tg4510 could be systematically altered and used to make new lines by pronuclear injection, the random integration process through which Tg4510 was established could never be precisely repeated, and any transgenic lines generated from these modified constructs would not adequately match the original Tg4510 line. We therefore first sought to establish a targeted-insertion equivalent to Tg4510 to which subsequent targeted-insertion lines expressing specific human tau variants could be precisely matched (an overview of the mouse lines generated and used is shown in Supplementary Fig. 1).

Although we find that mice from this targeted-insertion line (designated rT2/T2) overexpress even more tau_P301L_ than rTg4510, these mice exhibit significantly delayed overt atrophy and tau histopathology and an abnormal age-related flight response. Using whole-genome sequence analyses, we find that the transgene insertion/deletion (TgINDEL) mutation linked to the confounding effects in rTg4510 is an approximately 70-copy tau-transgene insertion in a ~250 kb deletion of the first exons and promoter regions of fbroblast growth factor 14 (Fgf14). Furthermore, we find that the tTA Tg-INDEL allele, which drives tau_P301L_ expression in both lines and is known to be sufficient to cause a more limited but progressive neuron loss, is an approximately 7-copy tTA-transgene insertion in a ~500 kb deletion that disrupts another five annotated genes (*Vipr2*-*Ptprn2*). Finally, we find that, in the absence of the *Fgf14* tau-TgINDEL, matching the high level of transgene overexpression in rTg4510 appears to be necessary to cause premature (≤7 months) tau histopathology, late-stage (>12 months) overt atrophy, and behavior abnormalities.

## Results

### TAU_P301L_ overexpression and gross forebrain atrophy

We used Flp/Frt recombination to target a single copy of the same tau_P301L_ transgene used to generate Tg4510 into mouse embryonic stem cells at an intergenic site downstream of collagen type I alpha I (Col1A1), a site previously demonstrated to promote transgene expression without dysregulating endogenous genes^7^. Mice with this single targeted *MAPT* cDNA transgene insertion are designated T2. In order to match the expression pattern in rTg4510 mice, these new T2 mice are crossed to the same tTA-driver line^5^ used to generate rTg4510 mice, resulting in rT2 mice. The rT2 mice are again crossed to T2 mice to generate mice homozygous for the tau_P301L_ transgene (i.e., rT2/T2, as shown in Supplementary Fig. 1).

**Fig. 1:**
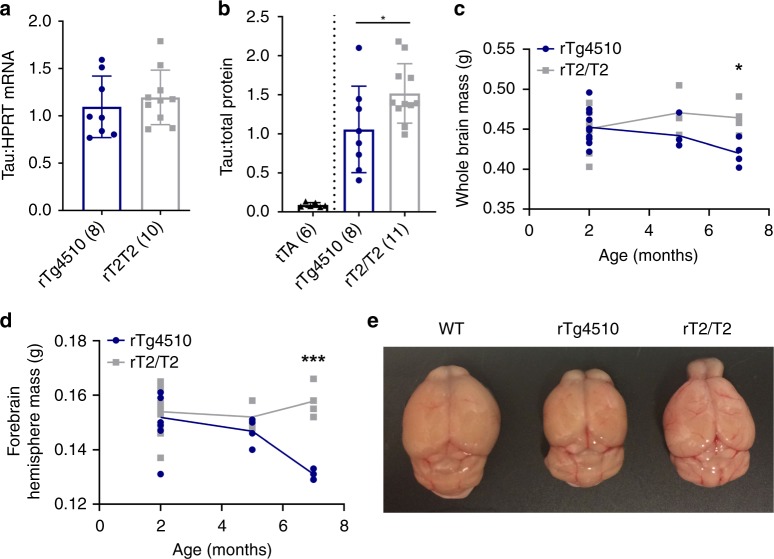
No premature gross forebrain atrophy in rT2/T2 despite greater overexpression of tau_P301L_. **a** We used relative qRT-PCR on RNA extracted from mouse forebrain-hemispheres to determine tau expression levels relative to *Hprt* (*n*’s in parentheses). Samples were run in triplicate and the experiment was replicated twice. All data are normalized to a positive calibrator run with each plate to control for inter-plate variability within experiments. An unpaired two-tailed *t* test was conducted (*p* = 0.501, *t* = 0.6882, d*f* = 16, 95% CI [−0.21 to 0.41]). **b** Protein from mouse forebrains was treated with phosphatase and analyzed by semiquantitative western blot relative to total protein loaded (*n*’s in parentheses). A pan-tau antibody (Tau46) was used to quantify fold-overexpression of human tau relative to endogenous tau in tTA mice, and was found to be 17-fold in rT2/T2 and 12-fold in rTg4510. An unpaired two-tailed *t* test revealed higher overexpression in rT2/T2 than rTg4510 (*p* = 0.046, *t* = 2.16, d*f* = 17, 95% CI [0.010–0.91]). The full blots are shown in Supplementary Fig. 13. **c** Whole brains were dissected from 2-month (rTg4510 *n* *=* 10, SD = 0.022; rT2/T2 *n* *=* 11, SD = 0.023), 5-month (rTg4510 *n* = 4, SD = 0.020; rT2/T2 *n* *=* 3, SD = 0.032), and 7-month-old mice (rTg4510 *n* *=* 4, SD = 0.017; rT2/T2 *n* *=* 4, SD = 0.020) and weighed. Bonferroni-corrected unpaired two-tailed *t* tests were conducted for 2-month (*p* = 2.56, *t* = 0.1894, d*f* = 19, 95% CI [−0.02 to 0.02]), 5-month (*p* = 0.58, *t* = 1.497, d*f* = 5, 95% CI [−0.08 to 0.02]), and 7-month (*p* = 0.046, *t* = 3.359, d*f* = 6, 95% CI [−0.08 to −0.01]) groups. **d** Forebrain-hemispheres were dissected from 2-month (rTg4510 *n* *=* 9, SD = 0.010; rT2/T2 *n* *=* 10, SD = 0.009), 5-month (rTg4510 *n* *=* 4, SD = 0.005; rT2/T2 *n* *=* 3, SD = 0.005), and 7-month (rTg4510 *n* *=* 3, SD = 0.002; rT2/T2 *n* *=* 4, SD = 0.006) old mice and weighed. A one-way ANOVA was conducted (*F*(5, 27) = 5.034, *p* = 0.0022) with Bonferroni’s multiple comparisons test for 2-month (*p* > 0.99, *t* = 0.31, d*f* = 27, 95% CI [−0.008 to 0.010]), 5-month (*p* > 0.99, *t* = 0.87, d*f* = 27, 95% CI [−0.010 to 0.021]), and 7-month (*p* = 0.0004, *t* = 4.42, d*f* = 27, 95% CI [0.011–0.0422]) groups. **e** Frozen brains from a 7-month female wild-type (left), rTg4510 (middle), and rT2/T2 (right) mouse were thawed and immersion-fixed in 10% formalin. Data in (**a**) and (**b**) are represented as group mean ± standard deviation. Data in (**c**) and (**d**) are represented as connected means. *p ≤ 0.05, ***p ≤ 0.001. Source data are provided as a Source Data file

We find that although rT2/T2 mice express the same levels of tau_P301L_ mRNA and even higher levels of protein in their forebrains than rTg4510 mice (Fig. 1a, b), rT2/T2 mice do not exhibit the dramatic premature loss of brain mass shown by rTg4510, which lose ~20% of their forebrain mass by 7 months of age (Fig. 1c, d). Gross forebrain atrophy, evident in rTg4510, is also absent in rT2/T2 at 7 months of age (Fig. 1e).

**Fig. 2:**
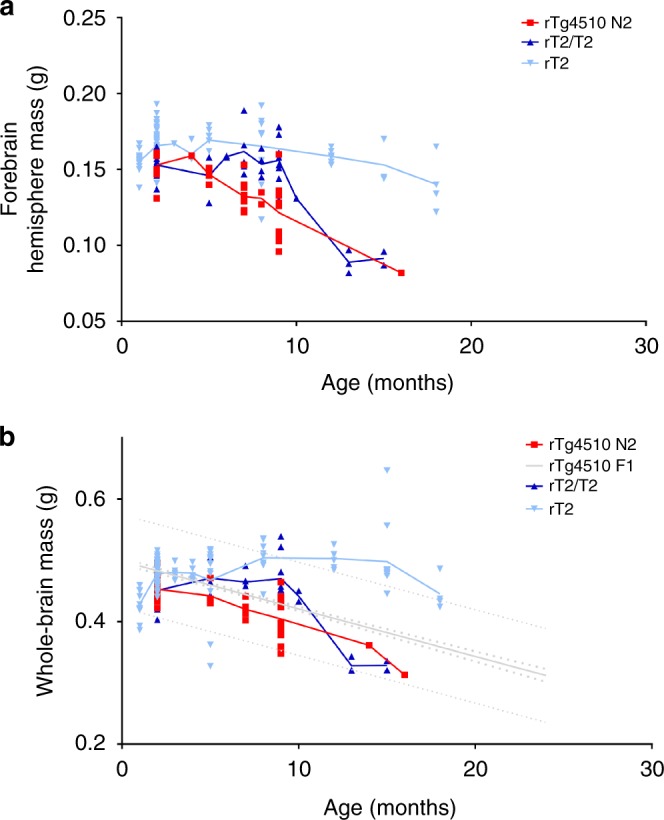
Pilot experiments with aged rT2 and rT2/T2. Forebrain-hemisphere **a** and whole brain **b** mass were measured for rT2/T2 (*n* *=* 32), rT2 (*n* *=* 120), rTg4510 F1 (FVB/129S6) (*n* *=* 554, accumulated data from Ashe lab), and rTg4510 N2 (FVB × FVB/129S6) (*n* *=* 32). Tissue samples were collected at varying ages, with limited numbers at the oldest ages (>12 months). Note that the genetic background of rTg4510 N2 is matched to rT2/T2 and that of rTg4510 F1 is matched to rT2. A comparison between rT2 and rT2/T2 reveals that severe loss in forebrain mass similar to rTg4510 is only observed in mice >12 months and only at the higher levels of transgene overexpression. Lines represent connected means. Thick dotted lines for rTg4510 F1 data represent 95% confidence bands while thin dotted lines represent 95% prediction bands. Source data are provided as a Source Data file

Our experimental design for the work described here included evaluating forebrain mass loss through 7 months, at which point rTg4510 mice exhibit severe forebrain mass loss and obvious gross forebrain atrophy (Fig. 1). Although the rT2/T2 mice do not share these phenotypes by 7M, we had sufficient animal numbers to continue these evaluations at 9 months, and then with fewer animals per time point at 10, 13, and 15 months (Fig. 2). The few rT2/T2 animals examined at 13 and 15 months in this pilot experiment each had severe forebrain mass loss and obvious gross forebrain atrophy that matched that of the few remaining background strain-matched rTg4510 examined at similar ages. We used rT2 mice as controls that incorporated the effects of the tTA TgINDEL but with more moderate overexpression of tau_P301L_. We did not find severe forebrain mass loss or obvious gross forebrain atrophy by even 18 months in the rT2 mice, although brain mass may be trending lower in the oldest rT2 mice evaluated. Expected rTg4510 brain mass values based on accumulated data from the Ashe lab are shown for comparison (Fig. 2b).

### TAU_P301L_-TgINDEL in Tg4510 disrupts *Fgf14*

**Fig. 3:**
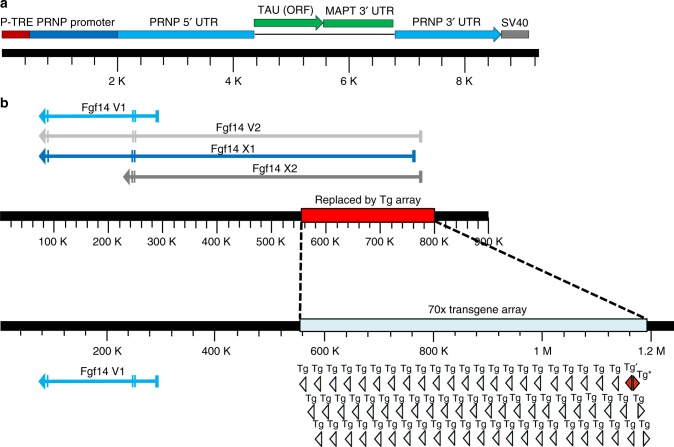
*Fgf14* is disrupted by a tau transgene array in Tg4510 mice. **a** Structure of the tau transgene monomer including the tetracycline response element (TRE) promoter, prion protein gene (*Prnp*) sequences, tau cDNA open reading frame (ORF), *Mapt* 3′ untranslated region (UTR), and SV40 polyadenylation signal. **b** Diagram of *Fgf14* mRNA splice variants and disruption by the transgene array. Vertical hashmarks in splice variants represent exons while arrows indicated the direction of synthesis. The red rectangle on the non-transgenic allele (top) represents the 243,608 bp deletion while the light blue rectangle on the transgenic allele (bottom) represents the approximately 70-copy insertion of the Tg multimer array. Tg, transgenes are light blue triangles, Tg′, partial transgene copy red triangle in the 3′→5′ orientation, Tg*, partial transgene copy red triangle in the 5′→3′ orientation

To determine if an insertion mutation caused by the tau_P301L_ transgene recombination into the Tg4510 mouse genome could explain the phenotypic difference between rTg4510 and rT2/T2, we performed whole-genome sequencing on DNA from a Tg4510 responder mouse using the Illumina HiSeq2500 High-Output system. Assembly and analyses of these reads revealed that approximately 70 head-to-tail copies of the tau transgene form an array that replaces a 243,608 bp region of *fibroblast growth factor 14* (*Fgf14*), the most telomeric gene on the long arm of chromosome 14 (Fig. 3b). Within the insertion array the transgene orientation switches from a 3′→5′ orientation to a 5′→3′ orientation, however, we are unable to determine precisely at which point in the array this switch occurs (Fig. 3b). We confirmed the transgene insertions sites by polymerase chain reaction (PCR) amplifying and resequencing key junctions and by performing fluorescence in situ hybridization (FISH) on Tg4510 genomic DNA (Supplementary Fig. 2c). The compiled tau transgene monomer sequence and all junction sequences have been submitted to GenBank.

### *Fgf14* expression is dysregulated in rTg4510 mice

**Fig. 4:**
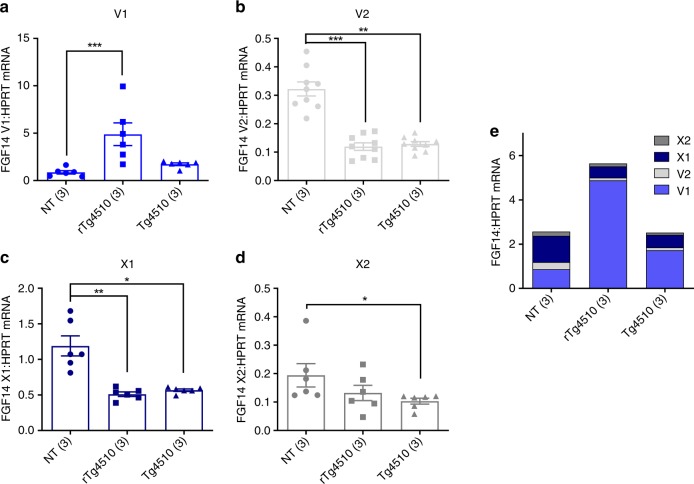
Dysregulation of *Fgf14* mRNA splice variants in rTg4510 and Tg4510 mice. Expression levels in the rostral one-third of dissected forebrain-hemispheres of *Fgf14* variants V1 (**a**), V2 (**b**), X1 (**c**), and X2 (**d**) in nontransgenic (NT), rTg4510, and Tg4510 mice (*n*’s in parentheses). Expression levels of each variant were determined by qRT-PCR relative to housekeeping gene *Hprt*. All samples were run in duplicate and data points from both experiments are shown. Kruskal–Wallis tests were performed for V1 (*H* = 13.2, d*f* = 2, *p* < 0.0001), V2 (*H* = 17.5, d*f* = 2, *p* < 0.0002), X1 (*H* = 12.3, d*f* = 2, *p* = 0.0001), and X2 (*H* = 6.9, d*f* = 2, *p* = 0.026) groups, followed by Dunn’s multiple comparisons tests. All data were normalized to a positive calibrator, and are expressed as the group mean ± standard error. **e** Compiled group mean data for all variants. *p ≤ 0.05, **p ≤ 0.01, ***p ≤ 0.001. Source data are provided as a Source Data file

Although transcription of *FGF14* has been reported to initiate at over unique 100 start sites^8^, at the time we began our analyses four representative splice variants of *Fgf14* were present in GenBank, and we restricted our analyses to these variants: V1 (NM_010201.4, encodes isoform 1a), V2 (NM_207667.3, encodes isoform 1b), X1 (XM_011244952.1), and X2 (XM_006518549.2). The deletion in Tg4510 removes the first 219 kb of V2 and terminates 266 kb upstream of the transcription start site for V1. Overall, this removes the promoters and first exons of variants V2, X1, and X2, leaving the coding region of only variant V1 intact (Fig. 3b). Available antibodies to Fgf14 protein do not distinguish between the products of these splice variants, and as a result Western blot analyses of Fgf14 differences between these lines was uninformative with respect to altered ratios of Fgf14 isoforms. We performed quantitative real-time PCR (qRT-PCR) of splice variants using RNA extracted from forebrain tissue of rTg4510, Tg4510, and nontransgenic (NT) mice and found that rTg4510 mice express 5.6-fold higher mRNA levels of variant V1 than NT mice and 2.8-fold higher levels of V1 than Tg4510 mice in their forebrains (Fig. 4a). Both rTg4510 and Tg4510 mice exhibit decreased expression of *Fgf14* variants V2, X1, and X2, presumably due to haploinsufficiency (Fig. 4b–d).

### tTA-TgINDEL in rTg4510 and rT2/T2 disrupts five more genes

**Fig. 5:**
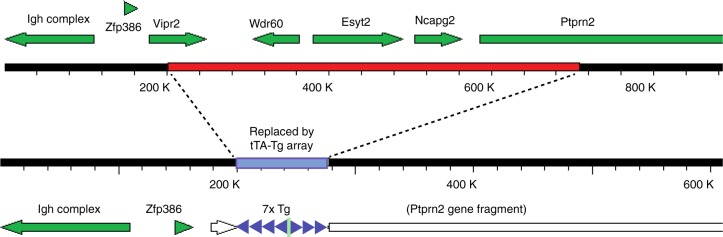
The tTA transgene array in rTg4510 and rT2/T2 mice disrupts genes on chromosome 12. Diagram of the uninterrupted chromosomal region and its disruption by the tTA-TgINDEL mutation. Tg, transgenes are blue triangles, cloning vector in inversion point of Tg array is a green line, uninterrupted genes are filled arrows, and partially deleted gene fragments are white arrows

We also performed whole-genome sequencing on DNA from an rT2/T2 mouse. We confirmed the expected configuration of the targeted tau_P301L_ transgene insertion in this line and also defined the tTA-TgINDEL mutation used to drive expression both in this line and in rTg4510. We found that approximately 7 head-to-tail copies of the tTA transgene form an array that replaces 508,119 bp in a gene-rich region of mouse chromosome 12 near the immunoglobulin heavy chain complex (Fig. 5). Similar to the configuration within the *Fgf14* tau-TgINDEL allele, the transgene orientation within the tTA insertion array switches from a 3′→5′ orientation to a 5′→3′ orientation, although in this instance a cloning vector DNA fragment is also incorporated at this inversion point. The massive genomic deletion in the tTA-TgINDEL removes the 3′ portion of *Vasoactive intestinal peptide receptor 2* (*Vipr2*), the 5′ portion of *protein tyrosine phosphatase, receptor type, N polypeptide 2* (*Ptprn2*), and encompasses all of the annotated genes *WD repeat domain 60* (*Wdr60*), *extended synaptotagmin-like protein 2* (*Esyt2*), and *non-SMC condensin II complex, subunit G2* (*Ncapg2*), as well as several predicted but uncharacterized genes (e.g., Gm20658). We confirmed the transgene insertions sites by PCR amplifying and resequencing key junctions (Supplementary Fig. 3). All junction sequences and the compiled tTA transgene monomer sequence, which includes a Ca^2+^-calmodulin kinase II (CaMKII) promoter fragment, have been submitted to GenBank.

### Transgene expression and *Fgf14*-TgINDEL accelerate pathology

**Fig. 6:**
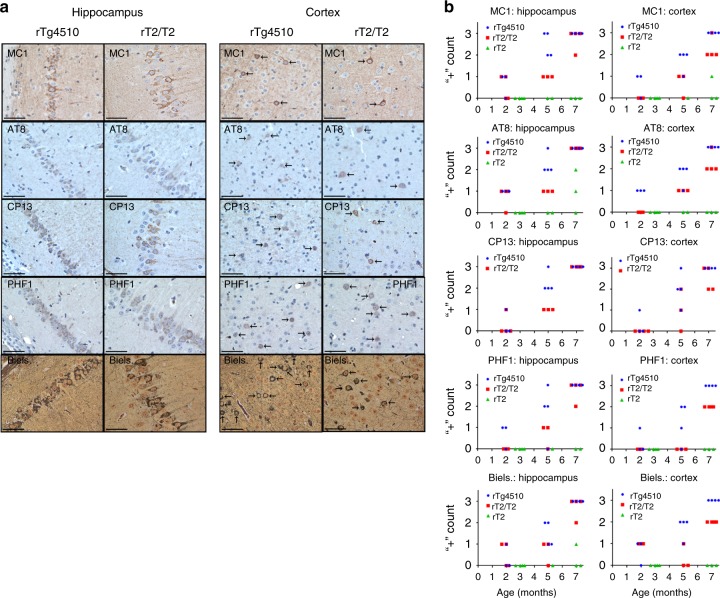
Comparison of tau histopathology in rT2, rT2/T2, and rTg4510. **a** Representative 40× images taken from somatomotor cortex and hippocampal CA1 of 7-month-old male rTg4510 and rT2/T2 mice. Stains used to visualize pathological tau aggregates included MC1, AT8, CP13, PHF1, and Bielschowsky (Biels.) silver stains, as indicated. Arrows indicate moderate to prominent positive labeling of tau deposits in cortex, while prominent labeling in hippocampus required no arrows because most or all of the cells exhibited positive labeling. Scale bars represent 50 µm. **b** Summary of semiquantitative analysis of tau histopathology progression in rTg4510 (*n* *=* 32M, 4 5M, 47 M), rT2/T2 (*n* *=* 42, 35, 47 M), and rT2 (*n* *=* 42, 25, 37 M) mice. Multiple linear regression analyses revealed significant regressions for every stain in both regions: AT8 cortex (*F*(3, 18) = 55.7, *R*^2^ = 0.89, *p* < 0.0001), AT8 hippocampus (*F*(3,18) = 25.8, *R*^2^ = 0.78, *p* < 0.0001), Biels. cortex (*F*(3,18) = 14.2, *R*^2^ = 0.65, *p* < 0.0001), Biels. hippocampus (*F*(3,18) = 17.3, *R*^2^ = 0.70, *p* < 0.0001), CP13 cortex (*F*(3,18) = 26.3, *R*^2^ = 0.78, *p* < 0.0001), CP13 hippocampus (*F*(3,18) = 40.6, *R*^2^ = 0.85, *p* < 0.0001), MC1 cortex (*F*(3,18) = 34.2, *R*^2^ = 0.83, *p* < 0.0001), MC1 hippocampus (*F*(3,18) = 24.0, *R*^2^ = 0.77, *p* < 0.0001), PHF1 cortex (*F*(3,18) = 18.3, *R*^2^ = 0.71, *p* < 0.0001), PHF1 hippocampus (*F*(3,18) = 17.2, *R*^2^ = 0.70, *p* < 0.0001). Also using multiple linear regression, a significant difference between rTg4510 and rT2/T2 *y*-intercepts was found for AT8 in the cortex (*p* = 0.017) and between rTg4510 and rT2/T2 progression of pathology (slope) for Biels. in the cortex (*p* = 0.024). A significant difference between rT2/T2 and rT2 *y*-intercepts was found for AT8 in the cortex (*p* = 0.014) and between rT2/T2 and rT2 progression of pathology in MC1 in the hippocampus (*p* = 0.0006), MC1 in the cortex (*p* = 0.002), AT8 in the cortex (*p* < 0.001), PHF1 in the hippocampus (*p* = 0.03), PHF1 in the cortex (*p* = 0.009), and Biels. in the hippocampus (*p* = 0.02). A significant difference between rTg4510 and rT2 progression of pathology was found for MC1 in the hippocampus (*p* = 0.0004), MC1 in the cortex (*p* = 0.001), AT8 in the cortex (*p* < 0.0001), PHF1 in the cortex (*p* = 0.0007), Biels. in the hippocampus (*p* = 0.004), and Biels. in the cortex (*p* = 0.0006). Source data are provided as a Source Data file

We evaluated human tau histopathology in tissue taken from the somatomotor cortex and hippocampal CA1 of 2-, 5-, and 7-month-old male and female rTg4510, rT2/T2, and rT2 mice using MC1, AT8, CP13, and PHF1 antibodies as well as a Bielschowsky (Biels.) silver stain. Semiquantitative analyses of stained sections (Supplementary Figs. 4–8) revealed similar pathological forms of tau in the first two of these three lines, although these accumulated at earlier ages and to a greater extent in rTg4510 than in rT2/T2 (Fig. 6b). Western blot densitometry of phosphorylated tau (pTau) showed higher signal in transgene-positive and transgene-negative control mice, but not between rTg4510 and rT2/T2, possibly due to variability in the data (Supplementary Figs. 9 and 10). However, analyses of insoluble high-molecular-weight tau was more consistent with the histopathology data (Supplementary Fig. 11). When we examined tissue samples from the rT2 line with less extreme tau_P301L_ overexpression (8.5× endogenous mouse tau level in rT2 vs. 17× in rT2/T2, see Supplementary Fig. 12) we found only limited instances of pathological-associated forms of human tau protein in a few tissue samples from 7-month-old mice (Fig. 6b).

### Age-related behavior changes in rT2/T2

**Fig. 7:**
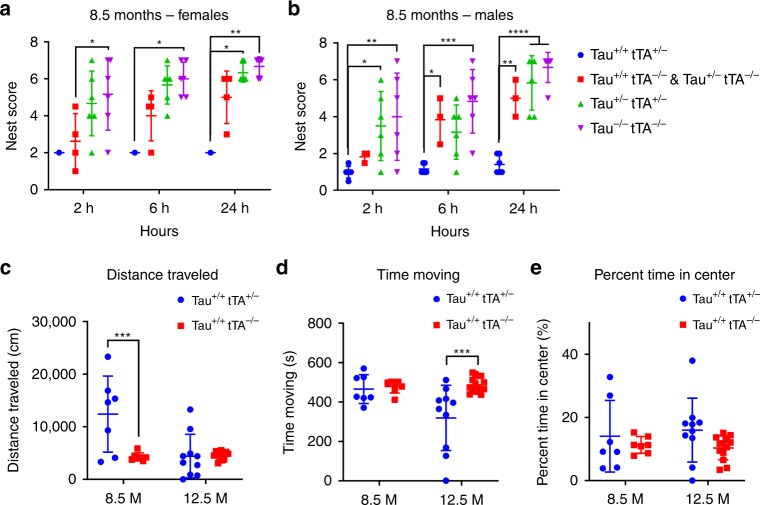
Nesting and open field behavior in rT2/T2 mice. **a**, **b** At 8.5 months of age, rT2/T2 (*n* = 7), rT2 (*n* = 12), and control littermates (Tau^+/+^tTA^−/−^ *n* = 7, Tau^−/−^tTA^−/−^ *n* *=* 12) were tested for their natural ability to construct a well-structured nest, which was scored 2, 6, and 24 h after presenting the mice with a fresh nestlet. An overall sex effect was detected indicating that females tended to build better nests than males. Therefore, each sex was analyzed separately. An impairment in nesting was observed in both male and female rT2/T2 mice, with rT2 mice performing significantly better than rT2/T2 mice by 24 h. For females (**a**), a two-way ANOVA revealed a significant main effect of genotype (*F*(3, 39) = 12.81, *p* < 0.0001). For males (**b**), a two-way ANOVA also revealed a significant main effect of genotype (*F*(3, 51) = 28.18, *p* < 0.0001). Tukey’s multiple comparisons test were run to compare genotypes within time points. **c**–**e** rT2/T2 and control littermates were tested in the open field at 8.5 months of age (*n* = 7 per group) and at 12.5 months of age (Tau^+/+^tTA^+/−^ *n* = 10, Tau^−/−^tTA^−/−^ *n* *=* 13). rT2/T2 mice exhibit signs of hyperactivity in that they travel significantly farther in the open field than controls at 8.5 months (two-way ANOVA: *F*(1, 33) = 9.395, main effect of genotype *p* = 0.0043), however, this declines to normal distance by 12.5 months (**c**). This shorter distance traveled at 12.5 months is reflected in less time spent moving (two-way ANOVA: *F*(1, 33) = 7.748, main effect of genotype, *p* = 0.0088) (**d**). rT2/T2 mice do not exhibit signs of anxiety, as they spend about the same amount of time in the center of the field as controls (**e**). Data represent mean ± standard deviation. *p < 0.05, **p < 0.01, ***p < 0.001, ****p < 0.0001. Source data are provided as a Source Data file

The Morris Water Maze has been regularly used to detect memory impairment in rTg4510 mice^1,2^, but 7-month-old rT2/T2 were too hyperactive in our hands to evaluate them in this task (see Fig. 7). We have not observed this high intensity flight response phenotype in mice in our rTg4510 colony. Nest building activity, which is a natural behavior in mice that has been used as a measure of behavior dysfunction in other mouse models of tauopathy^9^, was severely impaired in the rT2/T2 mice by 8.5 months whereas the rT2 mice exhibited normal nest building activity (Fig. 7a, b). Additional evaluation of the behavior phenotype in 8.5-month-old rT2/T2 mice using open field analysis suggest that these mice are hyperactive as indicated by distance traveled (Fig. 7c), but do not spend more time moving (Fig. 7d) and do not exhibit a higher level of anxiety (Fig. 7e). As these mice age (12.5 months), distance traveled is reduced, but apparently primarily due to significant reductions in time moving (Fig. 7c, d). We did not examine nest building activity or open field behavior in age-matched rTg4510 mice.

## Discussion

Of more than 300 therapeutic agents that had been reported by 2011 to effectively treat deficits in transgenic mouse models of Alzheimer’s disease, none had shown significant clinical benefit in human clinical trials^10^. An expert advisory panel of academic, industry, and government scientists convened to address this failure recommended in its best practices report “choosing models for preclinical studies that exhibit significant and well-characterized pathology relevant to the disease process of interest (that is, amyloid plaques, tau pathology, neuronal loss, oxidative stress/inflammatory changes, and so on).”^10^ Perhaps due in part to this recommendation, the rTg4510 mouse model of tauopathy, which exhibits profound premature neurodegeneration and NFTs, has become widely used for both basic and preclinical studies. Among models of tauopathy, rTg4510 mice display the most dramatic loss of neurons, and this loss progresses rapidly in young animals (2–7 months) (Fig. 1). However, we have now found that overexpression of the pathogenic tau variant in these mice is not sufficient to cause this premature and robust age-related loss of forebrain mass and gross forebrain atrophy, as originally and currently thought.

Two distinct mutant alleles are combined in rTg4510 mice: (1) the tau_P301L_-TgINDEL and (2) the tTA-TgINDEL needed to drive expression from the tau_P301L_ transgene allele. Both of these mutations were generated by pronuclear injection of synthetic mini-gene constructs into mouse single-cell embryos and were selected from among the many random integration events that occurred by screening for alleles that conferred the desired phenotype. In neither case, however, were the selected mutations themselves mapped or characterized. Our work presented here together with growing evidence from other groups indicates that the attractive rTg4510 tauopathy phenocopy found by screening for pathology similar to those found in human tauopathies arises from unintended dysfunctions conferred by the tau_P301L_ and tTA TgINDEL mutations.

Precisely defining the effects of the *Vipr2-Ptprn2* tTA-TgINDEL allele on the tauopathy phenocopy of rTg4510 is complex and in some ways problematic. The pronuclear injection experiment used to make this tTA allele, which is nominally expressed from a *CamKII* promoter fragment, generated lines with a wide array of expression patterns^5^ and the primary phenotype originally used to select this particular tTA-TgINDEL allele was functional tTA-driven overexpression selectively in mouse forebrain neurons^5^. Since genomic regulators retained in flanking sequences at each insertion site dictate the unique temporal and spatial expression pattern of tTA from each mutant allele, we were not surprised to find that four of the five annotated genes disrupted by this particular recombination event (*Vipr2*, *Ptprn2*, *Wdr60*, and *Esyt2*, Fig. 5) have prominent forebrain expression patterns (https://www.ncbi.nlm.nih.gov/gene/)^11^. We note that loss of both copies of the fifth annotated gene, encoding the chromosome condensin Ncapg2, results in embryonic lethality^12^. If nothing else, the unique tTA expression pattern generated from the combination of the *CamKII* promoter fragment within the context of the *Vipr2-Ptprn2* tTA-TgINDEL mutation defines the tau_P301L_ expression pattern in rTg4510, and likely defines the tissue specificity of the tauopathy phenocopy as well. Because we did not generate an alternative targeted insertion of a tTA transgene for our current work, we cannot determine if the loss of the genes disrupted by the *Vipr2-Ptprn2* tTA-TgINDEL directly affect the tauopathy phenocopy of rTg4510.

Because the tau transgene is expressed from a synthetic tTA-responsive promoter, tTA activity and tau expression are intrinsically linked, and as a result conclusively extricating the impacts of tau_P301L_ from those of tTA activity on phenotypes in rTg4510 may not be possible. The *Vipr2-Ptprn2* tTA-TgINDEL allele alone has been shown to be sufficient to cause a progressive neuron loss that leads to obvious degeneration of the dentate gyrus^13^. Suppression of tTA activity during the first 6 weeks of postnatal development with Dox has been shown to eliminate this observed neuron loss^13^, presumably by eliminating tTA binding and subsequent unintended activation of non-target gene expression at undefined loci in the genome. We note that the 490 + cognate tTA binding sites within the *Fgf14* tau-TgINDEL would also be expected to modify binding of excess tTA to and transcription activation at noncognate genomic sites, potentially complicating the interpretation of direct comparisons between rTg4510 mice and tTA-only littermates. For example, neuron loss in rTg4510 mice is less obvious in the dentate gyrus than in other regions of the hippocampus and cortex^1^, suggesting that the course of the documented tTA-driven neuronal toxicity and neuron loss is significantly altered in rTg4510 mice and may not contribute significantly to neuron loss. The design of the rTg4510 model precludes testing the alternative but not mutually exclusive hypotheses that overexpression of tau_P301L_ or tTA in this early developmental window is necessary to cause neuron loss. Administering Dox to rTg4510 mice from conception to 6 weeks, followed by removal of Dox resulted in robust tau histopathology by 54 weeks of age; neuron loss was reported to be absent, but data for neuron loss was not shown^14^. In addition, Dox suppression during development has been shown to result in a lower level of transgene overexpression in a model in which the *Vipr2-Ptprn2* tTA-TgINDEL allele drives expression of a CamKII target transgene^15^. If this is also true for expression from the *Fgf14* tau-TgINDEL, then the post-suppression level of transgene overexpression following Dox suppression during development^14^ may not be sufficient to drive neuron loss.

The fact that the *Vipr2-Ptprn2* tTA-TgINDEL allele is not genetically linked to the *Fgf14* tau-TgINDEL in rTg4510 has allowed us to use this same allele to drive expression in our new targeted-insertion rT2/T2 mice, and thereby matching the pattern of transgene expression and incorporating *Vipr2-Ptprn2* tTA-TgINDEL-linked confounding effects essentially identical to those found in rTg4510. Our new targeted-insertion rT2/T2 mice overexpress tau_P301L_ at levels even higher than in rTg4510 mice (Fig. 1a, b) from the same basic transgene construct (Fig. 3a), but only the rTg4510 mice exhibit robust age-related loss of forebrain mass and gross forebrain atrophy prior to one year of age (Fig. 1c–e). This result clearly demonstrates that a functional contribution from the *Fgf14* tau-TgINDEL mutation other than tau_P301L_ overexpression is a necessary cause of this premature overt atrophy. By comparing tau_P301L_ histopathology in tissue samples from rT2/T2 and rTg4510 mice (Fig. 6, Supplementary Figures 4–11), we are also able to conclude that confounding contributions from the *Fgf14* tau_P301L_-TgINDEL mutation accelerate the rate at which and the extent to which tau_P301L_ histopathology develops in rTg4510. Finally, we found that 7-month-old rT2/T2 mice in our hands develop an age-related high intensity flight response phenotype that prevented us from testing them in the Morris Water Maze, the task that has been used to measure memory impairment in rTg4510 mice^1,2^.

We found that the random recombination event that integrated the tau_P301L_ transgene array into the mouse genome in Tg4510 deleted 244 kb of the *Fgf14* gene (Fig. 3), and that this disruption results in the dysregulation of the ratios of *Fgf14* splice variants in mice that carry this mutation (Fig. 4). Unfortunately for anyone attempting to determine molecular mechanisms of tau-related dysfunctions using rTg4510, confounding dysfunctions in Fgf14 have the potential to impact almost every aspect of neurobiology, including synaptic transmission, plasticity, and neurogenesis^16^. The splice variants of the internal growth factor *Fgf14* differ in their N-terminal amino acid sequences, which contribute to their subcellular localization and their ability to regulate the voltage-gated sodium channels with which they interact^16–24^. The fact that unbiased transcriptome analyses of the extent of alternative promoter usage in adult human brains identified *FGF14* as one of the few outlier genes with more than 100 transcription start sites strongly suggests that differential regulation of this gene is critical for establishing and maintaining normal brain function^8^. The deletion of 244 kb of this gene in the *Fgf14* tau_P301L_-TgINDEL mutation would severely compromise the differential regulation of these functionally distinct *Fgf14* isoforms by removing a large percentage of these transcription start sites and alternative N-terminal coding sequences (Figs. 3 and 4). A translocation within a region of the human *FGF14* gene that is nearly identical to the syntenic segment of *Fgf14* disrupted by the tau transgene in rTg4510 has been reported to be linked to phenotypes ranging from ataxia (mother) to microcephaly and severe mental retardation (proband)^25^, suggesting that these types of *FGF14* disruptions may increase susceptibility to significant loss of brain mass and function.

Comparisons between the hemizygous rT2 line, which overexpresses tau_P301L_ at levels over 70% of that in rTg4510 mice (Supplementary Fig. 12), and the homozygous rT2/T2 line, with overexpression levels significantly higher than those of rTg4510, has allowed us to determine that high-transgene overexpression is a third critically important contributor to the tauopathy phenocopy in rTg4510 mice. By comparing tau_P301L_ histopathology in tissue samples from rT2 to those from rT2/T2 mice (Fig. 6b) we are able to conclude that pushing the level of tau_P301L_ overexpression to match or exceed that found in rTg4510 dramatically accelerates the rate and extent to which tau histopathology develops. Open field testing (Fig. 7) enabled us to quantify the hyperactivity phenotype observed in rT2/T2 mice and our results suggest that this phenotype is linked to the level of tau hyper- or over-expression in these models. These analyses revealed that the rT2/T2 mice traveled a greater distance than controls, but did not move more often, indicating that the true difference was the speed at which these mice moved. A similar increase in distance traveled in open field analysis was also seen in a mouse tauopathy model in which tau_P301L_ was overexpressed from an adeno-associated virus (AAV) vector, although in that model the increase in distance traveled paralleled a similar increase in time moving^26^. Similar to our findings with rT2/T2, the AAV1-tau_P301L_ overexpression model exhibited widespread accumulation of tau histopathology by 6 months without overt loss of neurons^26^. The overt atrophy that we did observe in limited numbers of aged rT2/T2 mice (>12 months), also appears to be a phenotype linked to hyperexpression of transgene, since we did not observe overt atrophy in aged rT2 mice (Fig. 2). We note that the overt atrophy that appears to develop in aged rT2/T2 mice is similar to the ages at which neuron loss is seen in another tauopathy mouse model^27^. Further studies with additional control lines (e.g., rT1 without the P301L mutation in the tau transgene) will be needed to conclude whether these impacts of transgene hyperexpression on phenotype in rT2/T2 are due specifically to extremely high levels of tau_P301L_ in particular, or to less specific cellular stresses caused by extreme overexpression of an exogenous gene in general. Our finding that high levels of tau_P301L_ overexpression directly leads to measureable dysfunctions in this protein product parallel findings in which overexpression of amyloid precursor protein (APP) causes additional phenotypes not found in models based on endogenous *App* expression levels^28,29^. Although massive overexpression of a transgene has been used as a means of inducing dysfunctions in many mouse models and is rationalized as accelerating dysfunctions that also might develop more slowly at physiological levels of expression, we need to remain cognizant of the fact that the mechanisms underlying these hyperexpression-induced dysfunctions may be unrelated to those that cause disease in patients.

Our work highlights the critical importance of identifying and using proper control lines in any experiments involving transgenic animal models. The classic controls for confounding contributions from the genomic position effects of randomly integrated transgenes has been to characterize populations of experimental lines (e.g., transgene with a mutation) and of control lines (e.g., transgene without a mutation), and then either continue to work with small populations of these lines (≥3 lines) with phenotypes that are representative of the average phenotypes of these characterized lines, or in most cases, with only single representative lines from each population. In almost every instance, lines with outlier phenotypes will have confounding contributions from their unique TgINDEL mutation. Although it may eventually be possible to reproduce the full range of premature tauopathy-like phenotypes found in rTg4510 by using targeted genomic modifications to generate control lines that more precisely and reproducibly generate the confounding contributions from the *Fgf14*-TgINDEL mutation (e.g., similar *Fgf14* dysregulation), this would be a new experimental system, and key findings from rTg4510 would need to be reproduced in this new system. Given the high cost in time and resources involved in making such a replacement, the onus of pursuing that work would have to fall on any group proposing to continue to use rTg4510 in their research. At this point, the targeted-insertion mouse models we are developing offers a system in which the *Fgf14*-TgINDEL mutation and the potential confounding contributions from this mutation are eliminated. If crossed to mice that carry the *Vipr2-Ptprn2* tTA-TgINDEL, as described here, these mice will still have confounding contributions from this activator allele, but this artificial environment would be matched in all mice in the series, with the only difference between lines being the particular tau variant specifically introduced into the targeted tau transgene. Nonetheless, mechanistic studies using our transgenic models would still need to account for possible contributions from the *Vipr2-Ptprn2* tTA-TgINDEL and from nonphysiological levels of transgene overexpression.

For more than a decade rTg4510 mice have been widely used as a model of human tauopathy but without recognizing the necessary confounding contributions from the *Fgf14* tau_P301L_-TgINDEL, and with only recent and inconsistent attempts to control for the contributions from the previously undefined *Vipr2-Ptprn2* tTA-TgINDEL allele^13^. We are explicitly not asserting that any hypotheses regarding tau pathophysiology generated from these studies using the rTg4510 model are incorrect. However, we believe that in all of these studies it is critical to account for possible contributions from the confounding effects that we have now defined. In particular, we caution against the use of rTg4510 for testing potential disease therapies, as we now know that a therapeutic agent could significantly improve or even eliminate tauopathy-like phenotypes in rTg4510 by incidentally correcting effects such as the contributions from the *Fgf14* tau_P301L_-TgINDEL mutation (i.e., as in rT2/T2) without modifying the intended drug target.

Although we have restricted our work to characterizing rTg4510, we believe that our findings are indicative of a much broader and deeper problem. Classic pronuclear injection technology generates mice in which a transgene construct is typically arrayed as multimers in unpredictable and irreproducible configurations (see Figs. 3 and 5) inserted in random locations into the mouse genome. Genomic position effects on transgene expression patterns as well as gene disruption and dysregulation caused by these random insert events invariably result in dramatic phenotypic variation between the mouse lines generated by this technology. All too often the mouse line selected for detailed characterization from among these many lines has been the line that exhibits the most “significant… pathology relevant to the disease process of interest”^10^, as was the case with rTg4510. Although such mouse lines are widely and routinely used, many of the TgINDEL mutations in these lines remain only marginally characterized at the genetic level. As we have shown here, advances in genomic DNA sequencing technology now allow us to fully define the TgINDEL mutations in these lines, enabling us to more accurately identify the confounding effects caused by these mutations and to account for their possible impacts on our work.

## Methods

### Animals

We generated ES cells with a Col1A1 “Flp-in” integration cassette by electroporating 25 µg of purified Col1a-frt-hygro-pA Plasmid (Addgene) into V6.5 Mouse Embryonic stem cells (C57BL/6× 129/sv) (Novus Biologicals NBP1–41162 passage 22). After G418 selection, Clone 15 was found to have integrated properly and had a perfect karyotype. All further FRT mediated targeting is to this ES modified cell line (V6.5Col1a#15). A construct was generated that was essentially identical to the construct used to generate Tg4510, but incorporated a Flp-In promoter cassette (PGK promoter-ATG-FRT), and 6 μg this construct and 0.5 µg pCAGGS-FlpE (Gene Bridges cat# A201) was transfected into the V6.5Col1a#15 ES cell line. Hygromycin selection at 140ug/mL was added day 2 through day 6, and Hygro resistant ES clones were picked on day 7. DNA was analyzed for 5′ (5ArmCol1A assay primer + TRE start Rev) and 3′ (AMP R Reverse + Hygro Connection) junctions, internal Tau (TAU assay F and R), and multiple integration assay (AMP R Reverse + TRE start R) by PCR. Southern blot was done for multiple integration confirmation using EcoRI digested genomic DNA and a 470 bp probe generated from the AmpR gene (Amp F + Amp R). Clone 6 was positive for all assays and the P301L mutation was verified, and this clone was expanded and karyotyped, and mice were generated by injection into blastocysts. These mice were back-crossed five times to FVB prior to generating the T2/T2 lines. ES Cell Assay Primer sequences: 5ArmCol1A pcr assay(5′-CAGGTGCACAGCATTGCGGACATG-3′), TRE Start Rev (5′-ATTGCTCCAGGCGATCTGAC-3′), Amp R Reverse(5-GGAATAAGGGCGACACGGAA-3′), Hygro Connecton (5′-ATCCACGCCCTCCTACATCGAA-3′). Tau Assay F (5′-GTTCGAAGTGATGGAAGATCACG-3′), and Tau Assay R (5′-TTGGGTGGAGTACGGACCA-3′). PCR Probe Primers sequences: Amp F (5′-CCTCCATCCAGTCTATTAATT-3′) and Amp R (5′-TCCTTGAGAGTTTTCGCCCCG-3′).

To generate tau-homozygous rT2/T2 mice from tau-hemizygous T2 mice, transgene-activated hemizygous males (CKTTA+/−Tau +/−) were bred to nonactivated hemizygous females (CKTTA−/−Tau+/−), resulting in tau homozygous progeny (Tau+/+). To maintain the rT2/T2 line, transgene-activated homozygous males (CKTTA+/−Tau+/+) were bred to nonactivated homozygous females (CKTTA−/−Tau+/+). Generation of rTg4510 mice, which utilizes an activator and responder system for transgene expression, has previously been described^2^. Briefly, a pTRE-prnp-tau plasmid was used to generate Tg4510 responders, which harbor a 0N4R human tau cDNA transgene regulated by a tetracycline response element (TRE). Activator mice harbor a tTA transgene under the control of the CaMKIIa promoter to drive expression specifically in forebrain excitatory neurons. Tau expression is activated in bigenic rTg4510 progeny of an activator-responder cross. For the rTg4510 line, responder Tg4510 were maintained on a FVB/N background while activator mice were maintained on a 129S6 background. To match the genetic background to that of rT2/T2 mice, we increased the amount of FVB/N and used these mice to compare expression levels in rTg4510 and rT2/T2 mice. To generate these mice, responder Tg4510 mice were maintained on a FVB/N background while activator mice were maintained on a mixed 129S6 and FVB/N background. Nontau-expressing transgenic littermates were used as controls. Both male and female mice were used, and were combined in statistical analyses after demonstrating the absence of significant gender effects (*P* > 0.05). All experiments with animals described in this study were approved by and conducted in full accordance with the American Association for the Accreditation of Laboratory Animal Care and the Institutional Animal Care and Use Committee at the University of Minnesota.

### qRT-PCR

mRNA expression levels of each Fgf14 variant were quantified relative to a reference gene, *Hprt*. All Fgf14 primers (Supplementary Table 1) were designed to span at least partially unique regions of each variant. Total cellular RNA was extracted from homogenized forebrain tissue using RNeasy Lipid Tissue Kit (Qiagen) according to the manufacturer’s instructions. RNA samples were treated with DNaseI (New England Biolabs) to digest contaminating DNA, and subjected to cDNA synthesis using the iScript cDNA synthesis kit (Invitrogen) according to the manufacturer’s instructions. PCR reactions were set up in a 20-µl volume in 96-well plates, with 2–3 replicates per sample. SYBR Green PCR master mix (Roche) was used and reactions were run in the LightCycler^®^ 480 instrument (Roche) (Supplementary Table 2). A final melting curve confirmed that single amplicons were present for each variant and reference reactions, and a basic relative quantification was performed using the ∆∆C_T_-Method (LightCycler^®^ 480 Software release 1.5.0 SP3). All data were normalized to a positive-calibrator sample used in each experiment.

### Protein extraction and phosphatase treatment

Total protein was extracted from mouse forebrain-hemisphere tissue in radioimmunoprecipitation assay (RIPA) buffer (50 mM tris-HCl, 150 mM NaCl, 1 mM EDTA, 0.5% Triton X-100, 1% sodium deoxycholate, 0.3% sodium dodecyl sulfate (SDS), 0.1 mM phenylmethyl sulfonyl fluoride, 0.2 mM 1,10-phenoanthroline Monohydrate, Phosphatase Inhibitor Cocktail A (Sigma), Protease Inhibitor Cocktail (Sigma), Phosphatase Inhibitor Cocktail 2 (Sigma)). Homogenates were nutated and centrifuged at 15,700 × *g* for 90 min at 4 °C and the supernatant was collected^31^.

To obtain RIPA-insoluble, sarkosyl-insoluble fractions, a modified version of a previously published method was used^31^. RIPA-insoluble pellets were homogenized in 1% sarkosyl and incubated at room temperature for 30 min with constant shaking. Samples were centrifuged for 1 h at 100,000 × *g* at 20 °C, and the supernatant and pellet were separated and diluted in O+ buffer (62.5 mM tris-HCl, pH 6.8; 10% glycerol; 5% 2-mercaptoethanol; 2.3% SDS; 1 mM EGTA; 1 mM EDTA; 1 mM PMSF; 1 mM Na_3_VO_4_; 1 mM NaF; 10 μl/ml of protease inhibitor cocktail P8340; Sigma-Aldrich). Samples were boiled for 3 min and stored at −20 °C.

For treatment with calf intestinal alkaline phosphatase (CIP, New England Biolabs), samples were resuspended in 10 µl CIP buffer (100 mM NaCl, 50 mM Tris-HCl, 10 mM MgCl2, 1 mM dithiothreitol, EDTA-free protease inhibitor cocktail, pH 7.9) per 1 µg protein. One unit CIP per µg protein was added to the samples prior to incubation at 37 °C for 30 min. Samples were then concentrated using Amicon Ultra centrifugal filters (Millipore).

### Western blot and analysis

For sarkosyl-insoluble fractions, total protein concentration was normalized according to pellet weights. Total protein concentrations for all other samples were determined by Pierce^™^ Bicinchoninic Acid protein assay (Thermo Scientific). Equal amounts of protein for each sample were loaded and separated using SDS-PAGE on 10%, 10.5–14%, or 10–20% tris-HCl gels (Bio Rad). Protein was transferred to nitrocellulose membranes (Bio Rad), which were blocked with 5% bovine serum albumin (Sigma) in 1× tris buffered saline buffer with Tween 20 (TBST) buffer (10 mM Tris-Base (Sigma), 0.2 M NaCl (Macron Chemicals), 0.1% Tween-20 (Sigma) pH 7.4). Protein was immunoblotted with Tau46 (Cell Signaling Technology #4019, dilution 1:10,000), Tau13 (BioLegend #MMS-520R, dilution 1:60,000), GAPDH(14C10) (Cell Signaling Technology #2118, dilution 1:4000), GAPDH(GA1R) (Thermo Scientific #MA5-15738, dilution 1:5000), AT8 (Thermo Scientific #MN1020, dilution 1:1000), anti-human tau (Abcam # ab74391, dilution 1:10,000), and βIII-tubulin (ProSci #79–720, dilution 1:10,000) antibodies. Additional antibodies from Peter Davies for phospho-epitopes on tau included MC1 (dilution 1:800), CP13 (dilution 1:1,000), and PHF1 (dilution 1:1,500). To visualize antibody immunoreactivity using a LiCor imaging system and Image Studio software, IRDye-linked goat anti-mouse 800CW and goat anti-rabbit 680LT secondary antibodies were used (LI-COR Biosciences,dilution 1:100,000). Following LiCor image acquisition using Image Studio software (Odyssey), Amido black staining solution (Sigma-Aldrich) was used for total protein quantification according to manufacturer’s instructions. Immunoreactivity and Amido black staining were quantified by densitometry using OptiQuant version 3 software, following guidelines for total protein quantification^30^.

### Immunohistochemistry and analysis

Mouse brain hemispheres were immersion-fixed in 10% formalin for 48 h before processing. Unstained sagittal TMA sections (4 µm) were deparaffinized and rehydrated using standard methods. Bielschowsky silver staining was performed using standard techniques. For antigen retrieval, slides were incubated in 6.0 pH buffer (Reveal Decloaking reagent, Biocare Medical, Concord, CA) in a steamer for 30 min at 95–98 °C, followed by a 20 min cool down period. Subsequent steps were automated using an immunohistochemical staining platform (Nemesis, Biocare). Endogenous peroxidase activity was quenched by slide immersion in 3% hydrogen peroxide solution (Peroxidazed, Biocare) for 10 min followed by TBST rinse. A serum-free blocking solution (Rodent Block M, Biocare Medical, Concord, CA) was placed on sections for 20 min. Blocking solution was removed and slides were incubated in primary antibody diluted in 10% blocking solution/90% TBST. Mouse monoclonal antibodies from Peter Davies were applied at the following dilutions: CP13 1:1000, MC-1 1:800 and PHF1 1:1500, mouse monoclonal PHF-Tau: clone AT8 (Thermo Scientific #MN1020) 1:1000. Sections were incubated in primary antibody for 60 min at room temperature followed by TBST rinse and detection with biotinylated anti-mouse secondary (Vector Laboratories #BP-9200, dilution 1:200) for 30 min followed by a TBST rinse. After the rinse, SA-HRP (Biolegend #405210, RTU) was applied for 30 min. All slides then proceeded with TBST rinse and detection with diaminobenzidine^32^(Covance, Dedham, MA). Slides were incubated for 5 min followed by TBS rinse then counterstained with CAT Hematoxylin (Biocare, Concord, CA) for 5 min. Slides were then dehydrated and coverslipped. Images were gathered using an Axioskop microscope (Zeiss, Germany) at 40× magnification and a PixeLINK microscope camera (PL-A623C) with PixeLINK Capture SE software version 2.2 (Firewire camera release 4, Copyright^©^ 2000–2006). Adobe Photoshop CS2 version 9.0 was used to match the color of different images of the same histological stain. Semi-quantitative analysis of images was conducted using a “+” system^1^. A blinded observer gave scores for three sections per sample indicating severity of pathology using “−” for no positive labeling, “+” for occasional positive labeling, “++” for moderate positive labeling, “+++” for prominent positive labeling. To summarize these results, the number of “+” signs was counted for each animal and region, and multiple linear regression analyses were conducted using R statistical programming language to test for differences between rTg4510, rT2/T2, and rT2.

### Whole-genome sequencing and sequence analyses

Genomic DNA was extracted from a non-activated Tg4510 mouse harboring the 0N4R human tau transgene using the DNeasy Blood & Tissue kit (Qiagen) according to the manufacturer’s instructions. Genomic DNA was analyzed by nanodrop and agarose gel to verify the quality (O.D. 260/280 ratio > 1.8) and quantity (300 ng for library construction).

Sequencing was performed on Illumina HiSeq 2500 High-Output system using rapid SBS chemistry at the University of Minnesota Genomics Center, Minneapolis, MN. Following quality control, a TruSeq Nano DNA library was prepared from the genomic DNA sample and was sequenced on a single Illumina lane to generate 2 × 125 bp paired-end reads. The average insert length in the library was 350 bp.

All sequence analyses were conducted using the University of Minnesota’s installation of the Galaxy web-based suite of software^33^. We isolated individual sequence reads that spanned the end of the transgene using the BLAT alignment tool^34^ and mapped paired sequence reads to the transgene sequence using Bowtie2^35^. We screened these data sets and identified unmapped reads in which the paired read mapped to the transgene sequence and then further analyzed these screened sets, in part using the Integrative Genomic Viewer^36^, to find the genomic insertion points and a single 5′−5′ transgene fragment junction. Bowtie2 mapping of the sequence data to the *Fgf14* genomic or *Vipr2-Ptprn2* data (as appropriate) indicated that roughly half as many reads mapped to the unique sequences between the insertion points as mapped outside of this region, indicating that the Tg4510 mice have only a single copy of this portion of the *Fgf14* genomic sequence. To estimate transgene copy number, we used sets of closely linked SNPs to distinguish between the PrnP sequence in the transgene and that in the FVB genome^37^, and found that the dataset contains more than 35-fold more reads generated from transgene PrnP sequences than from the diploid mouse PrnP sequence on chromosome 2, indicating that more than 70 copies of transgene sequence are inserted at the *Fgf14* locus in the Tg4510 mice. Similar analyses were performed using polymorphisms in the CamKII genomic data to determine the copy number in the tTA transgene array. Sequences mapped to the reference Tg sequence were assemble into a consensus transgene sequence using SPADES^38^.

### PCR and sequencing

Selected regions of Tg4510 and rT2/T2 genomic DNA were amplified by PCR using Herculase II Fusion DNA polymerase (Agilent Technologies). For Tau transgene junctions in Tg4510 DNA, PCR and nested PCR reactions were run (Supplementary Tables 3–5). CaMKIIα-tTA transgene junctions were confirmed using rT2/T2 DNA (Supplementary Tables 6 and 7). Gel bands were isolated from a low melting point agarose gel (NuSieve GTG, Lonza) and DNA was purified following digestion with β-agarase (New England Biolabs). Gel-purified PCR products were sequenced with classical Sanger sequencing at the University of Minnesota Genomics Center, Minneapolis, MN.

### Mouse cell culture and metaphase slide preparation

Spleen from a Tg4510 mouse (28th generation backcross onto FVB genetic background) was minced into a single-cell suspension and cultured for 24–28 h with 5 µg/ml Concavalin A (mouse T-cell mitogen). Cultures were exposed to colcemid (Irvine Scientific) for 15 h overnight followed by harvest using standard cytogenetic protocols. Metaphase spread slides were prepared from methanol-acetic acid fixed cell pellets and FISH was performed the following day.

### Fluorescent in situ hybridization

DNA probes derived from the tau transgene sequence were labeled by nick translation reaction (Nick Translation Kit—Abbott Molecular) using Orange 552 dUTP (Enzo Life Science), ethanol precipitated and resuspended in hybridization buffer. The probe/hybridization buffer mix and slide were denatured, probe was applied to the metaphase slide, and slide was hybridized for 24 h at 37 °C in a humidified chamber. After hybridization, the FISH slides were washed in a 2× SSC solution and counterstained with DAPI stain to enable chromosome identification by G-band patterning. Fluorescent signals were visualized on an Olympus BX61 microscope workstation (Applied Spectral Imaging, Vista, CA) with DAPI and Texas Red filter sets. FISH images were captured using an interferometer-based CCD cooled camera (ASI) and FISHView ASI software.

### Behavioral experiments

For nesting experiments, animals were not disturbed for at least 24 h prior to testing. Each animal was placed in its own clean cage with a new nestlet placed in the center of the cage in the morning. Nests were photographed and scored 2, 6, and 24 h after the start of the test. Scores of 0–7 were given as follows^9^: 0—nestlet untouched, 1—<10% of nestlet was shredded, 2–10–50% of nestlet shredded but no shape to nest (flat), 3–10–50% of nestlet shredded and there is shape to nest, 4–50–90% of nestlet shredded but no shape to nest (flat), 5–50–90% of nestlet shredded and there is shape to nest, 6—>90% of nestlet shredded but no shape to nest (flat), 7—>90% of nestlet shredded and nest had walls that were at least as tall as the mouse on 50% of the sides. Reported scores are the average of two individual blinded scorers.

Open field testing was done two days after the conclusion of nesting experiments. Animals were not disturbed for at least 24 h prior to testing and were placed in the testing room for 30 min prior to testing. The Open field setup was a plastic tub with opaque white walls, measuring 15 in wide by 18.5 in long by 12 in high. The arena floor was covered with new, prescented cage bedding. Each mouse was released in the center of the arena and allowed to freely explore for 10 min. All trials were monitored using a computerized tracking system (Noldus Ethovision XT 10.0; Noldus Information Technology). All animals were tested in the morning to avoid activity differences from time of day.

### Statistical analysis

Analyses were conducted using GraphPad Prism version 6.00 software (GraphPad Software) and R statistical programming language. Gender differences were detected only in nest-building behavioral assays and analyses were conducted accordingly. In all cases, *p* < 0.05 was considered to be statistically significant.

### Availability of unique biological materials

We will not be able to distribute mouse ES cells, since they will not tolerate repeated passaging, but all other materials and mouse lines will be available for distribution when requested.

### Reporting summary

Further information on research design is available in the Nature Research Reporting Summary linked to this article.

## Supplementary information

Supplementary Information

Reporting Summary

Source Data

## Data Availability

Sequence data that support the findings of this study (e.g., Figs. 3 and 5, and Supplementary Figs. 2 and 3) have been deposited in GenBank with the primary accession codes MF989990 (Tg4510 transgene), MF989991 (head-to-tail transgene junction), MF989992 (head-to-head transgene junction), MF989993 (transgene array—*Fgf14* promoter region junction), and MF989994 (transgene array—*Fgf14* intron junction). Accession numbers for tTA-TgINDEL sequences are MK674482 (CamKII-tTA transgene_monomer), MK674483 (CamKII-tTA Tg-Vector-Tg junction), MK674484 (CamKII-tTA_Tg-Ptrn2_junction), MK674485 (Vipr2-CamKII-tTA Tg junction), MK674486 (CamKII-tTA Tg head-to-tail junction Type 1), MK674487 (CamKII-tTA Tg head-to-tail junction Type 2), MK674488 (CamKII-tTA Tg head-to-tail junction Type 3). The source data underlying Figs. 1a–d, 2a, b, 4a–e, 6b, and 7a–e and Supplementary Figs. 9b, 10b, 11, and 12a, b are provided as a Source Data file.
