# Supplementary material for: Factors other than hTau overexpression that contribute to tauopathy-like phenotype in rTg4510 mice
Source: Nat Commun. 2019 Jun 6;10:2479. doi: 10.1038/s41467-019-10428-1 (PMC6554306; doi:10.1038/s41467-019-10428-1)
Supplement: Supplementary file 1 — Supplementary Information [file 41467_2019_10428_MOESM1_ESM.pdf]

Supplementary Information for

"Factors other than hTau overexpression that contribute to tauopathy-like phenotype in rTg4510 mice"

Gamache et al.

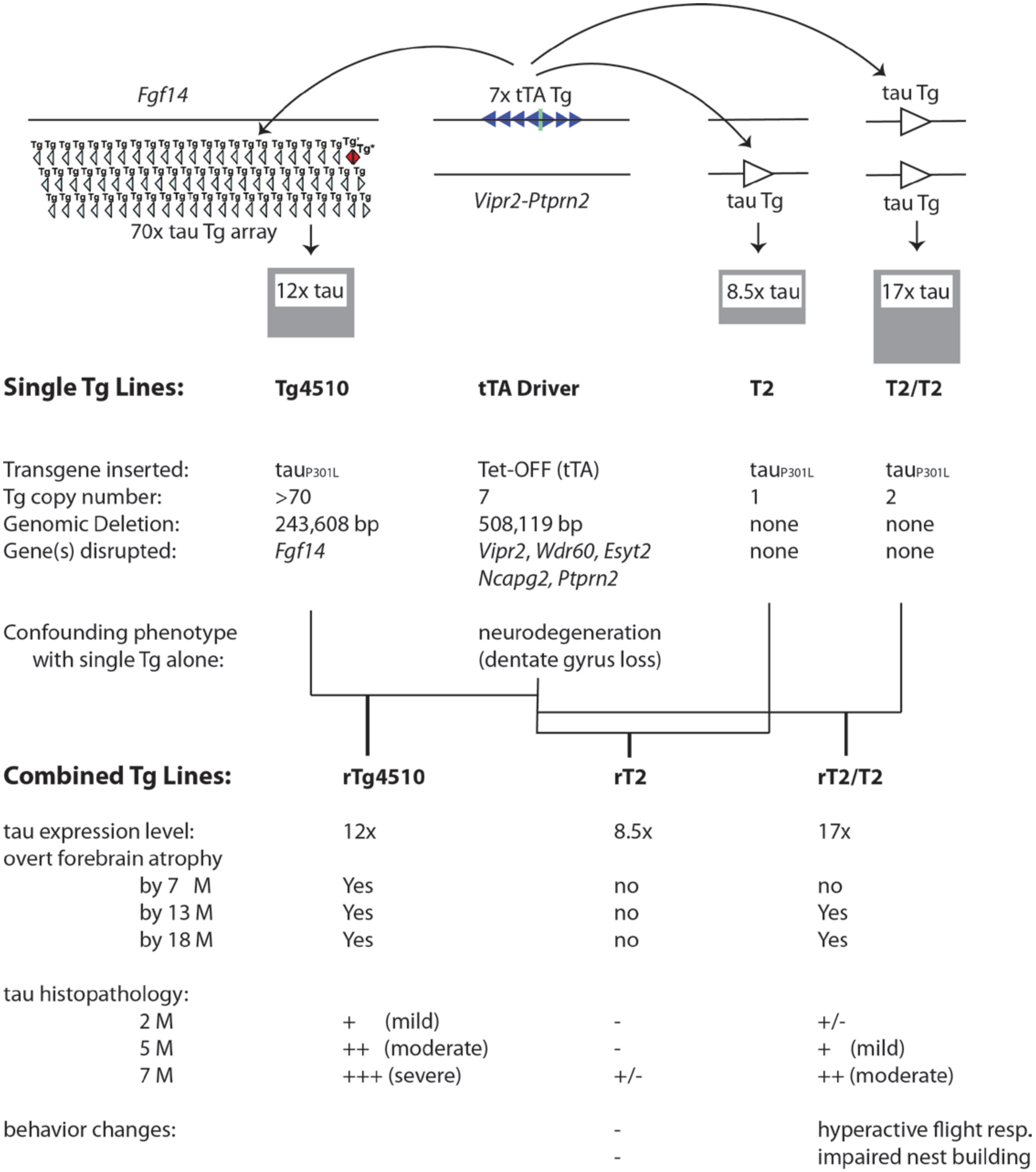

Supplementary Figure 1. Overview of mouse lines and key phenotypic differences. The tau$_{P301L}$ transgene array in Tg4510 and the tTA (Tet-Off) transgene array in the expression driver line, both of which replace large segments of the mouse genome, can only be maintained as hemizygous alleles, as depicted.

The single-copy targeted insertion of the same tau_{P301L} transgene generated for the work described here does not disrupt any endogenous genes and was used as either a hemigyous (T2) or homozygous (T2/T2) allele, as shown. Expression from the tau_{P301L} transgene requires the tTA transcription activator protein from the driver transgene, and bi-transgenic lines that carry both the driver and responder transgenes are designated as rTg4510, rT2 or rT2/T2 ("r" = regulatable). Our work demonstrates that the *Fgf14* tau-TgINDEL mutation is necessary to cause the rapidly progressive tauopathy phenocopy in rTg4510 (rTg4510 vs rT2/T2), and that key hallmarks of this phenocopy do not develop without extremely high levels of tau_{P301L} overexpression (rT2 vs rT2/T2). The *Vipr2-Ptprn2* tTA-TgINDEL allele alone without the tau_{P301L} transgene is sufficient to cause significant neurodegeneration, and contributions from this allele must be controlled for in any line using this allele.

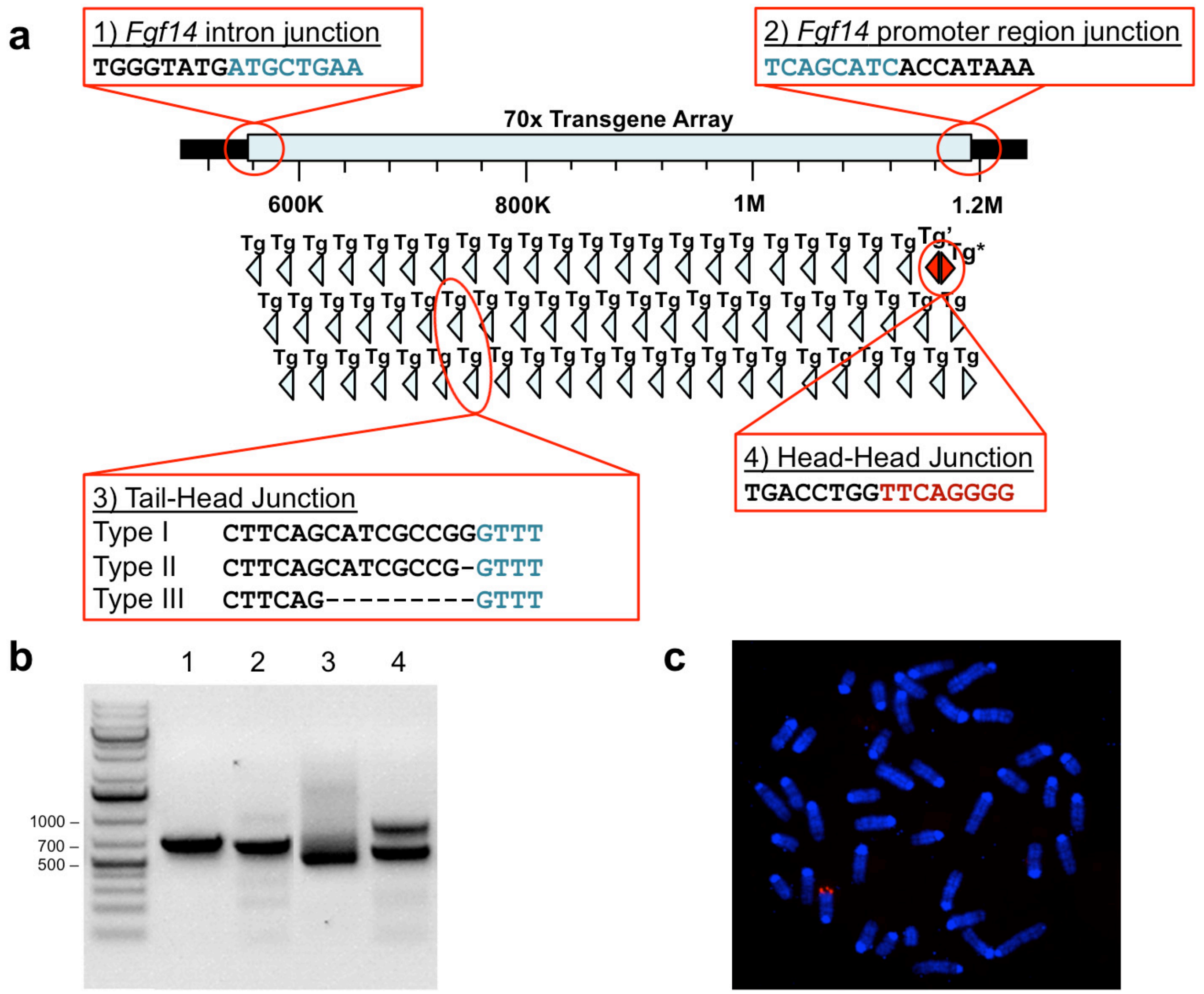

Supplementary Figure 2. Confirmation of tau-TgINDEL transgene insertion sites and array junctions in the Tg4510 genome. (a) Diagram of the following key junctions: 1) the junction between the transgene array and the *Fgf14* intron, 2) the junction between the transgene array and the *Fgf14* promoter region, 3) Tail-Head junctions between adjacent transgene copies (which occurred in three Types), and 4) the Head-Head junction at the point in the array in which the transgene flips orientation. Note that the transgene copies at the *Fgf14* intron, *Fgf14* promoter region, and Head-Head junctions are truncated in different places and therefore the portions of transgene sequence shown do not match. (b) Gel electrophoresis of PCR products shown in panel *a*. The *Fgf14* intron junction amplicon is 811 bp, the Tail-Head junction amplicon is 685 bp, the Head-Head junction amplicon is 626 bp, and the *Fgf14* promoter region junction amplicon is 767 bp. (c) Metaphase chromosomes from a Tg4510 mouse were subjected to FISH, using DNA probes derived from the tau

transgene sequence tagged with an Orange 552 fluorescent label. Fluorescent signal is detected near the telomere on the long arm of chromosome 14, identified by its unique G-band patterning.

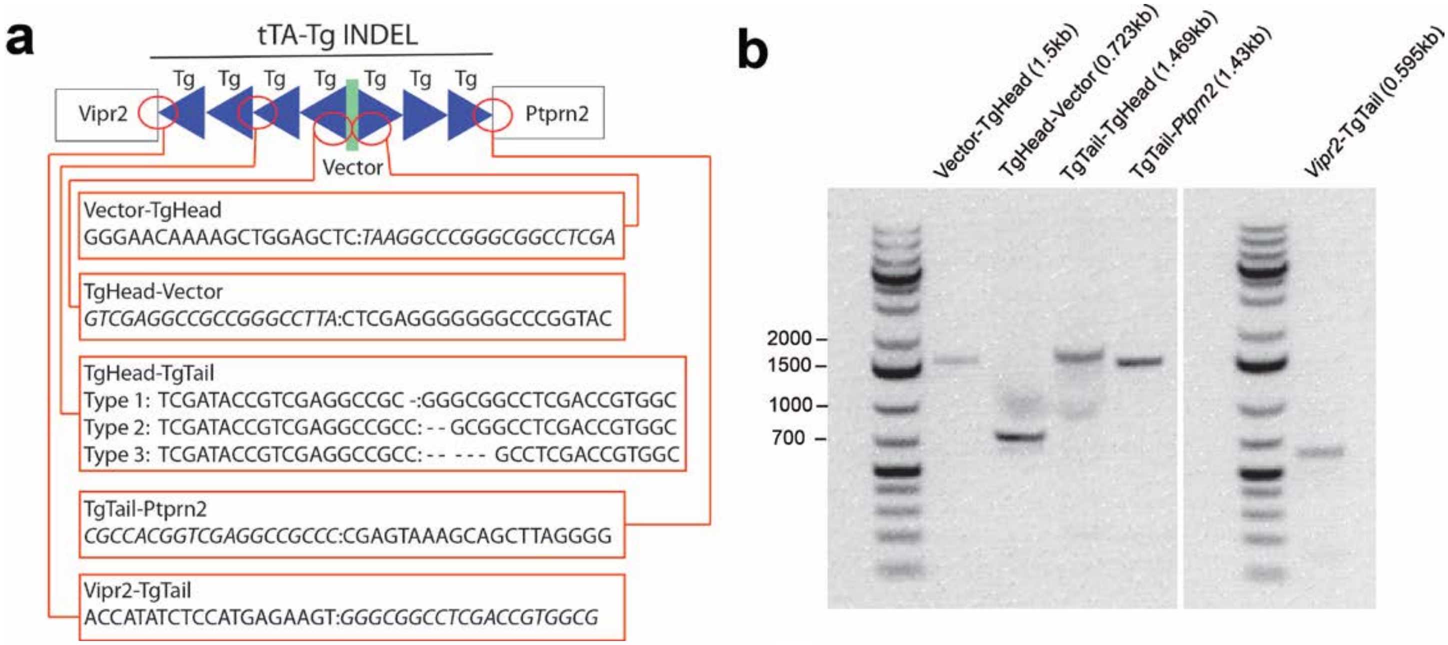

Supplementary Figure 3. Confirmation of the junctions in the tTA-TgINDEL allele. (a) Diagram of the following key junctions: 1) the junction between the transgene array and *Vipr2*, 2) the junction between the transgene array and *Ptprn2*, 3) Tail-Head junctions between adjacent transgene copies (which occurred in three Types), 4) TgHead-Vector, and 5) vector-TgHead. (b) Gel electrophoresis of PCR products shown in panel *a*.

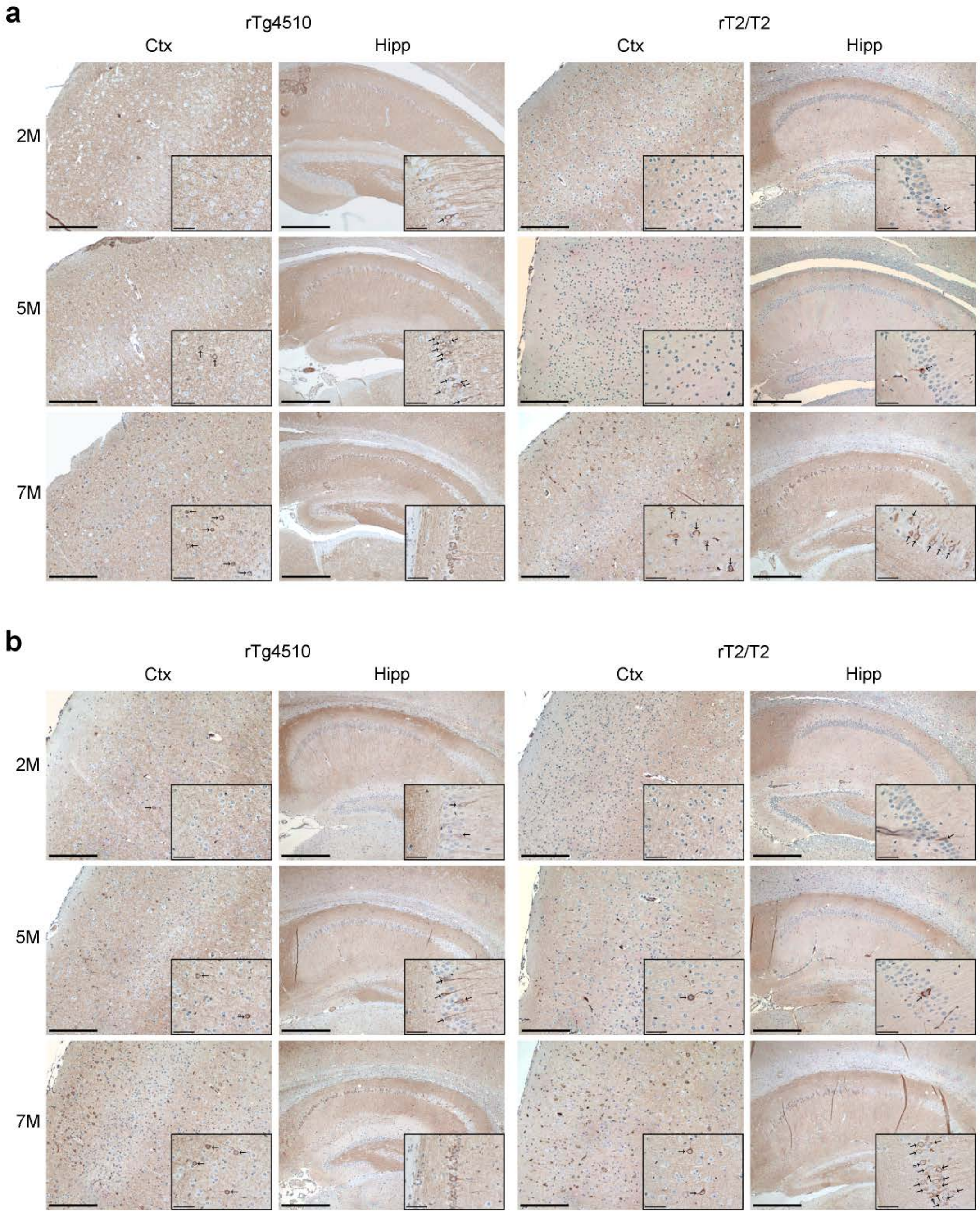

Supplementary Figure 4. Accumulation of MC1-positive neurons progresses more slowly in rT2/T2 than in rTg4510 brains. MC1 (Tau aa 7-9 and 326-330) antibody was used to detect an early-stage pathological change in tau conformation in the hippocampus (Hipp) and somatomotor areas of cortex (Ctx) of 2-month, 5-month, and 7-month old female (a) and male (b) rTg4510 and rT2/T2 mice. Insets of hippocampal images show the CA1 region. MC1-positive tau deposits in rTg4510 brains appear smaller than in rT2/T2 brains due to neuronal shrinkage. Scale bars in low magnification images represent 250 µm in hippocampus and 200 µm in cortex. Scale bars in high magnification insets represent 50 µm.

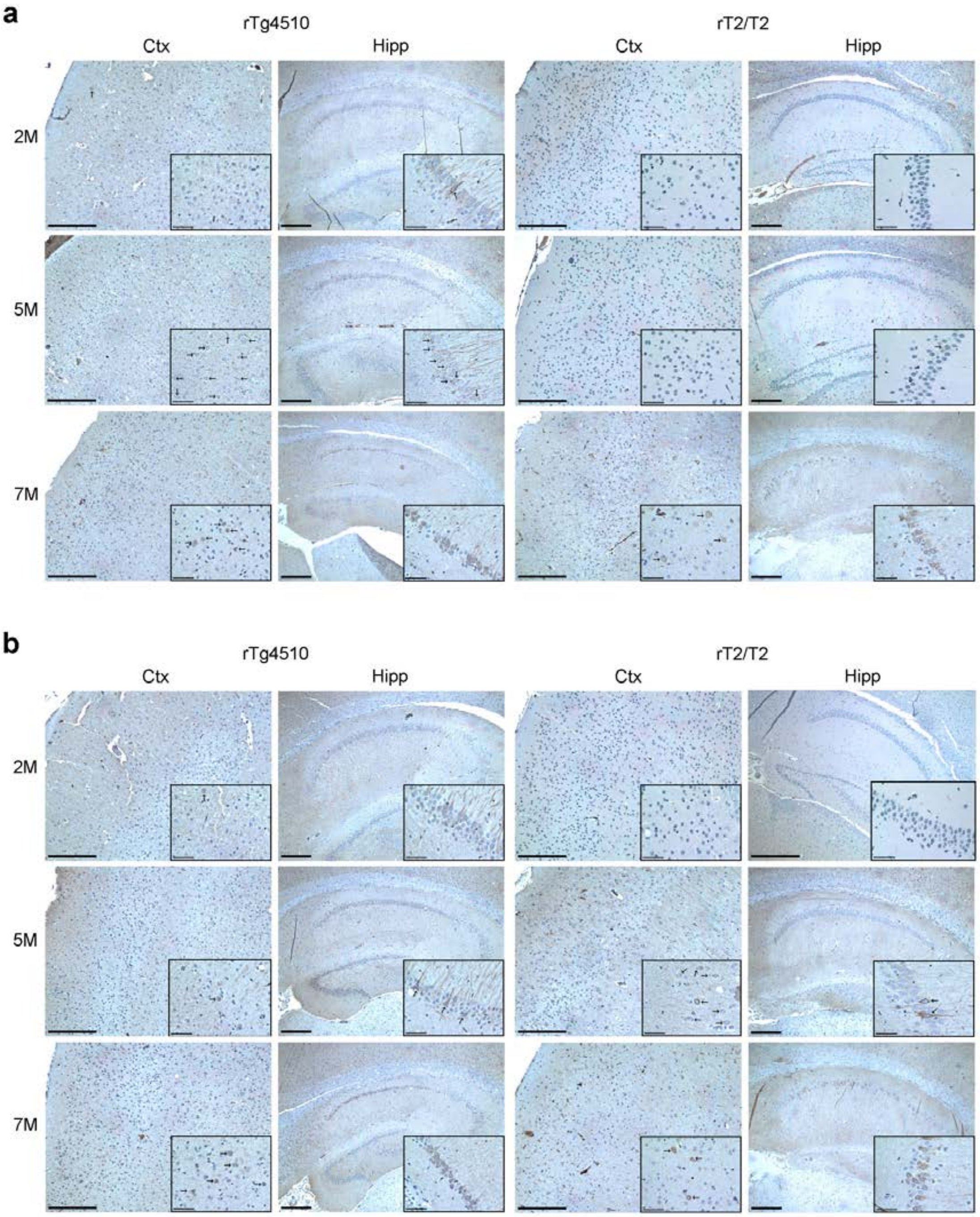

Supplementary Figure 5. Accumulation of CP13-positive neurons progresses more slowly in rT2/T2 than rTg4510 brains. CP13 (Tau pSer^{202}) antibody was used to detect an early-stage pathological phosphoepitope on tau in the hippocampus (Hipp) and somatomotor areas of cortex (Ctx) of 2-month, 5-month, and 7-month old female (a) and male (b) rTg4510 and rT2/T2 mice. Insets of hippocampal images show the CA1 region. CP13-positive tau deposits in rTg4510 brains appear smaller than in rT2/T2 brains due to

neuronal shrinkage. Scale bars in low magnification images represent 250 µm in hippocampus and 200 µm in cortex. Scale bars in high magnification insets represent 50 µm.

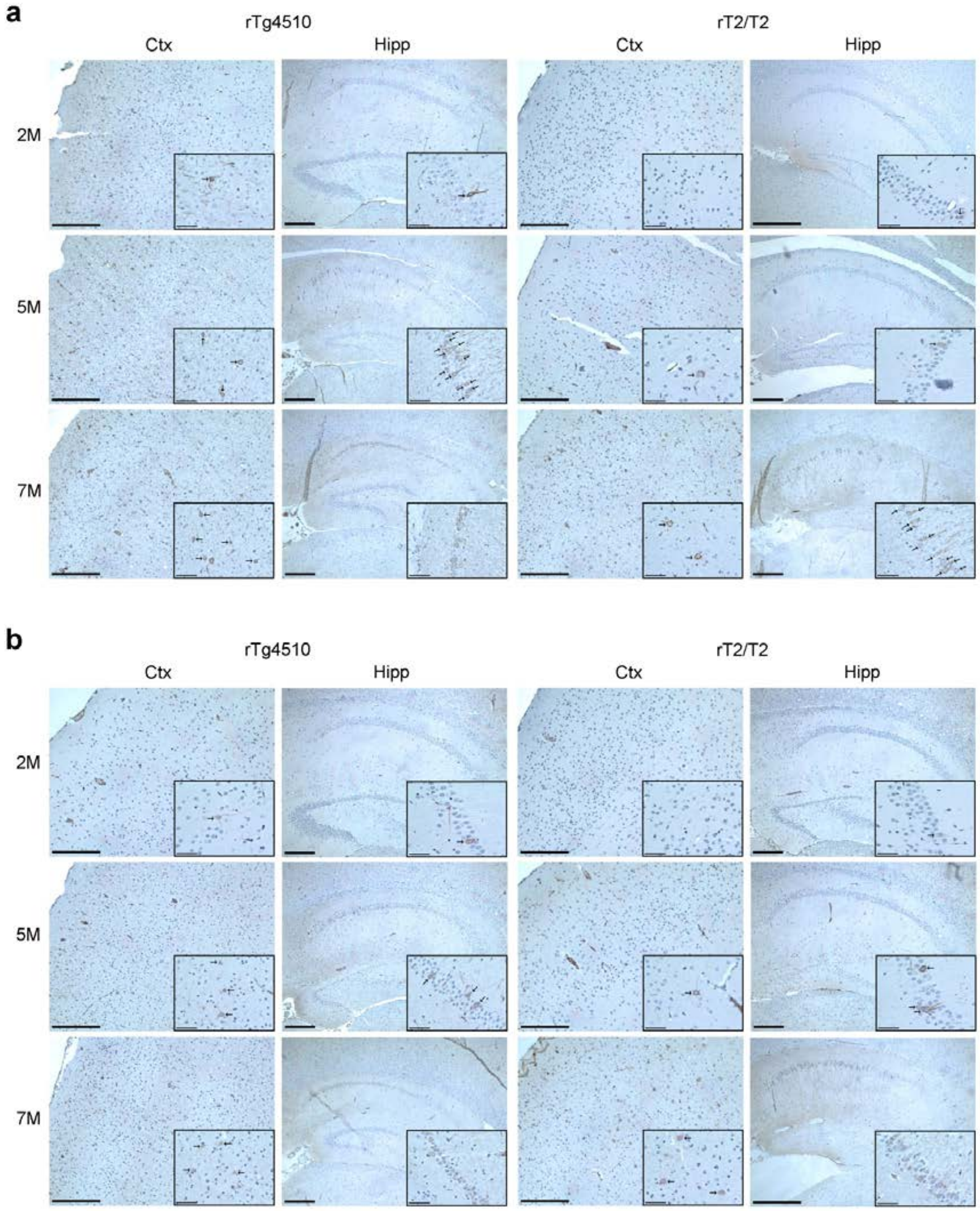

Supplementary Figure 6. Accumulation of AT8-positive neurons progresses more slowly in rT2/T2 than rTg4510 brains. AT8 (Tau $pSer^{202}/pThr^{205}$) antibody was used to detect a pathological pre-tangle phosphoepitope on tau in the hippocampus (Hipp) and somatomotor areas of cortex (Ctx) of 2-month, 5-month, and 7-month old female (a) and male (b) rTg4510 and rT2/T2 mice. Insets of hippocampal images show the CA1 region. AT8-positive tau deposits in rTg4510 brains appear smaller than in rT2/T2 brains due to neuronal

shrinkage. Scale bars in low magnification images represent 250 µm in hippocampus and 200 µm in cortex. Scale bars in high magnification insets represent 50 µm.

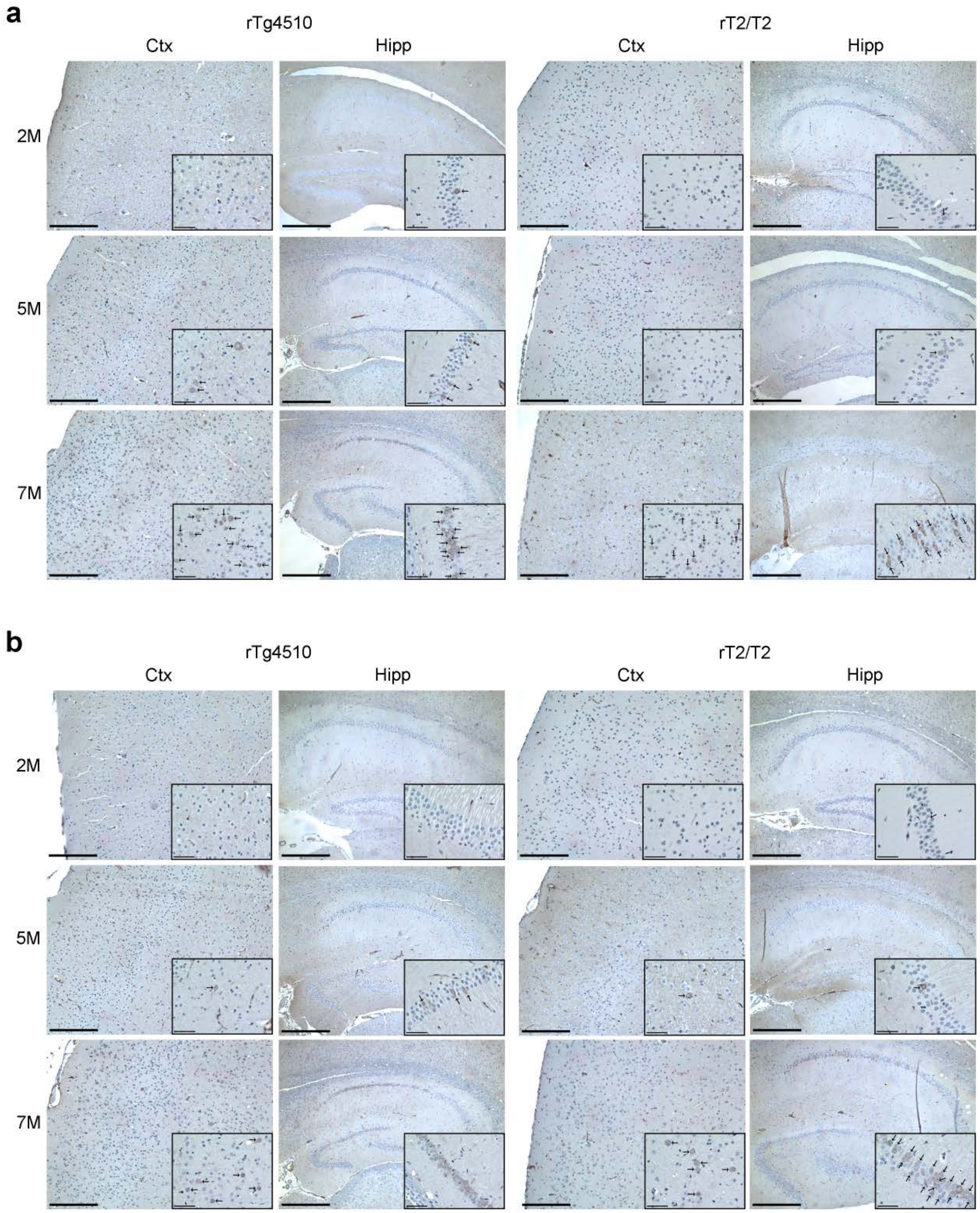

Supplementary Figure 7. Accumulation of PHF1-positive neurons progresses more slowly in rT2/T2 than rTg4510 brains. PHF1 (Tau pSer^{396}/pSer^{404}) antibody was used to detect a late-stage pathological phosphoepitope on tau in the hippocampus (Hipp) and somatomotor areas of cortex (Ctx) of 2-month, 5-month, and 7-month old female (a) and male (b) rTg4510 and rT2/T2 mice. Insets of hippocampal images show the CA1 region. PHF1-positive tau deposits in rTg4510 brains appear smaller than in rT2/T2 brains due to neuronal shrinkage. Scale bars in low magnification images represent 250 µm in hippocampus and 200 µm in cortex. Scale bars in high magnification insets represent 50 µm.

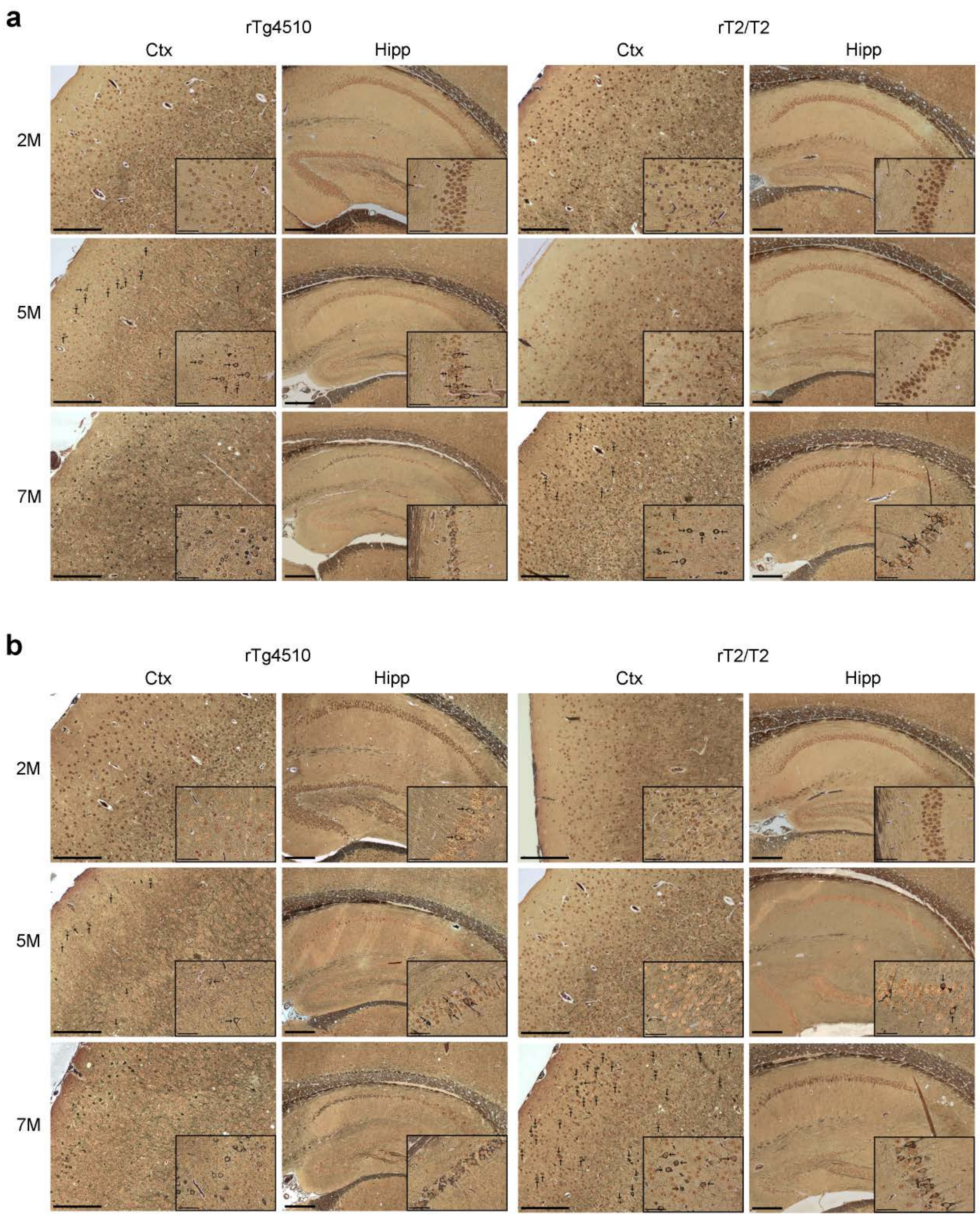

Supplementary Figure 8. Accumulation of neurofibrillary tangle-positive neurons progresses more slowly in rT2/T2 than rTg4510 brains. Bielschowsky silver stain was used to detect mature neurofibrillary tangles in the hippocampus (Hipp) and somatomotor areas of cortex (Ctx) of 2-month, 5-month, and 7-month old female (a) and male (b) rTg4510 and rT2/T2 mice. Insets of hippocampal images show the CA1 region. Deposits in rTg4510 brains appear smaller than in rT2/T2 brains due to neuronal shrinkage. Scale bars in low magnification images represent 250 µm in hippocampus and 200 µm in cortex. Scale bars in high magnification insets represent 50 µm.

a

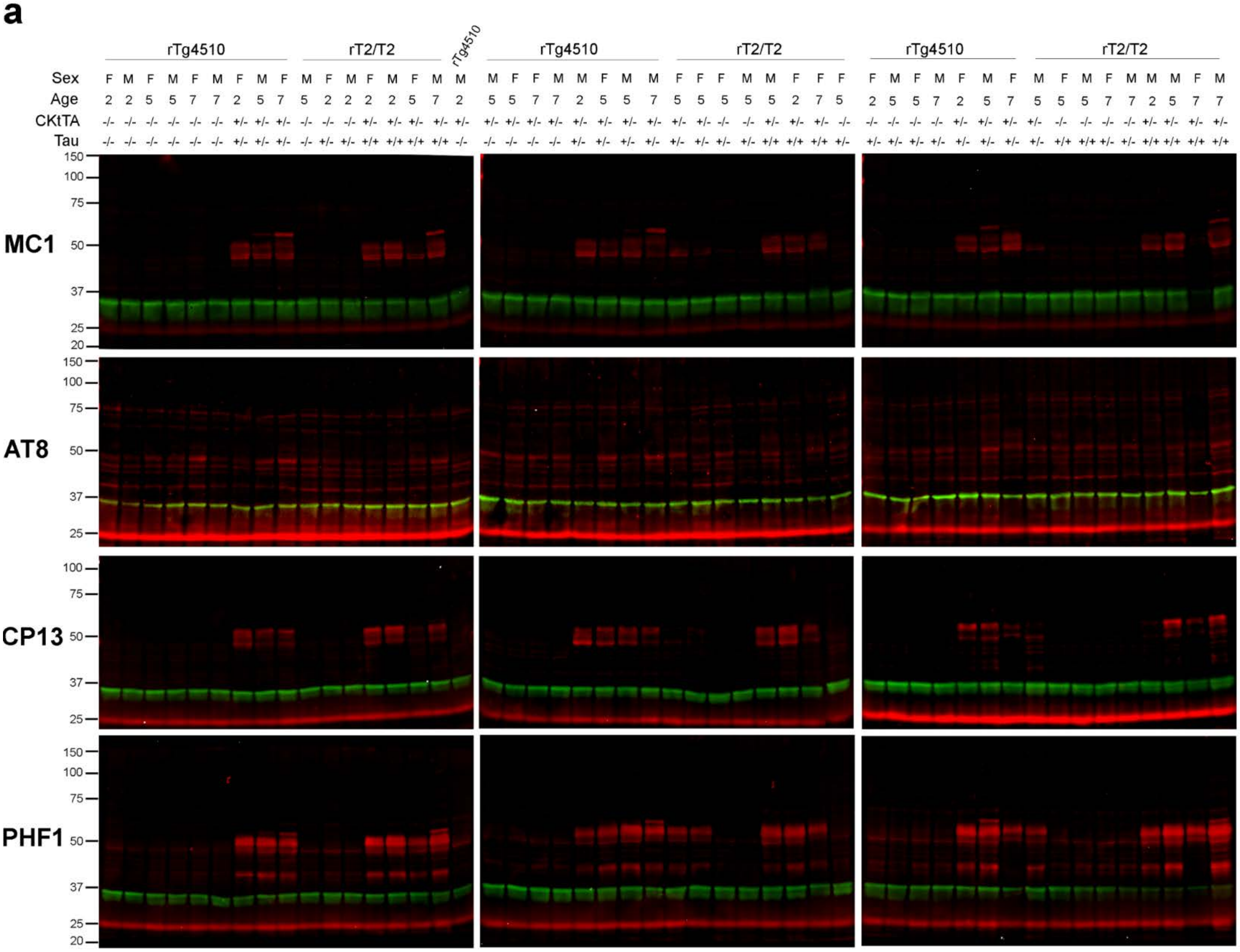

rTg4510
rT2/T2
rTg4510
Sex
Age
CKtTA
Tau
MC1
AT8
CP13
PHF1
150
100
75
50
37
25
20

b

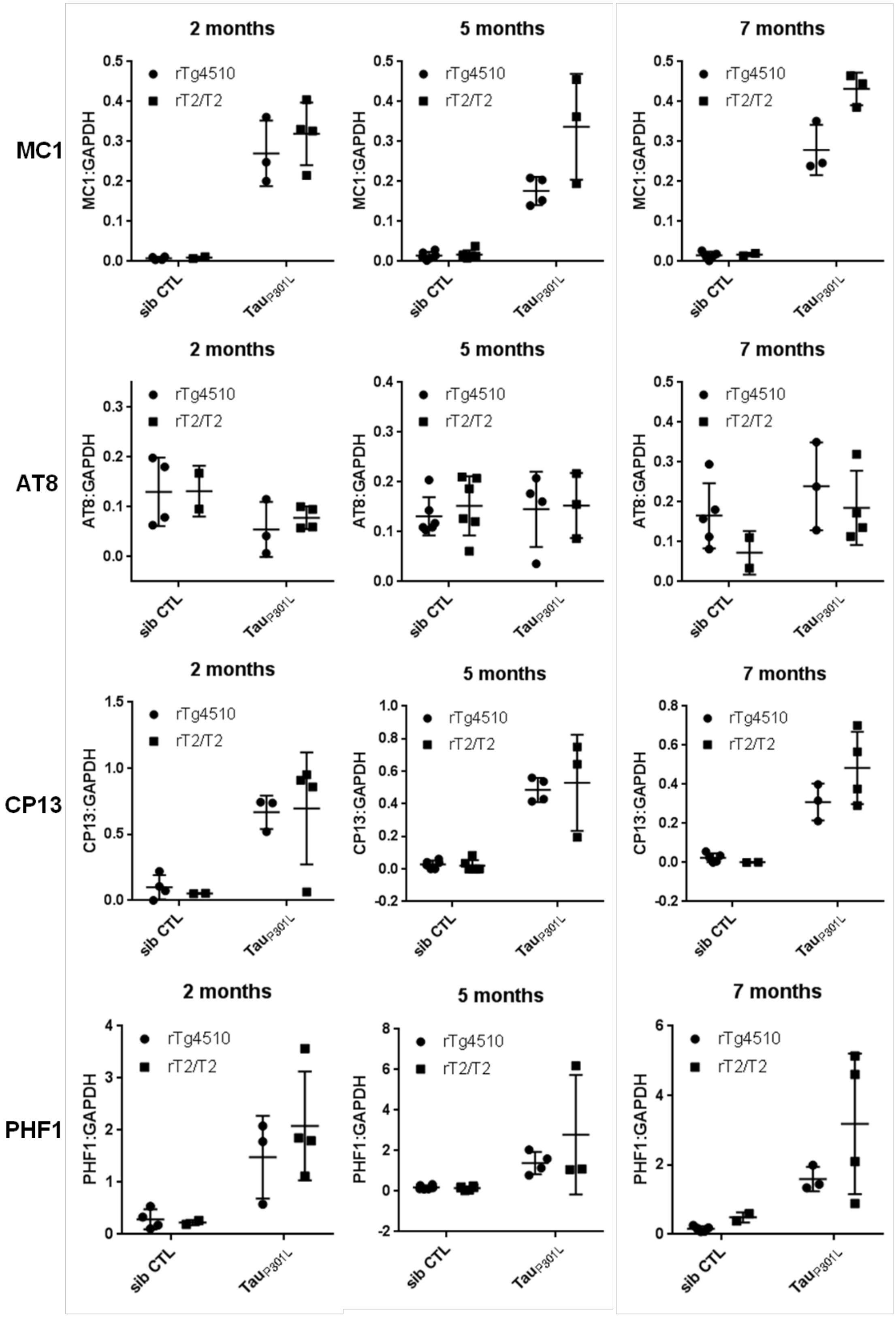

Supplementary Figure 9. Quantification of pathological tau in rTg4510 and rT2/T2 forebrains relative to GAPDH. Western blots (a) and densitometric quantification (b) of pathological Tau relative to GAPDH (14C10)

levels in rTg4510 (*n* = 3 2M, 4 5M, 4 7M), rT2/T2 (*n* = 4 2M, 3 5M, 4 7M), and sibling controls (rTg4510 *n* = 4 2M, 6 5M, 5 7M; rT2/T2 *n* = 2 2M, 6 5M, 2 7M). (a) Antibodies for pathological Tau included MC1 (Tau aa 7-9 and 326-330), AT8 (Tau $pSer^{202}/pThr^{205}$), CP13 (Tau $pSer^{202}$), and PHF1(Tau $pSer^{396}/pSer^{404}$). Sibling controls showed little to no positive labeling, and the AT8 antibody gave a weak signal. Tau-hemizygous rT2/T2 littermates were excluded from all analyses. (b) Mann-Whitney tests were conducted to compare Tau_{P301L}–expressing rTg4510 to rT2/T2 and revealed no significant differences for any age or any immunolabeling. Source data are provided as a Source Data file.

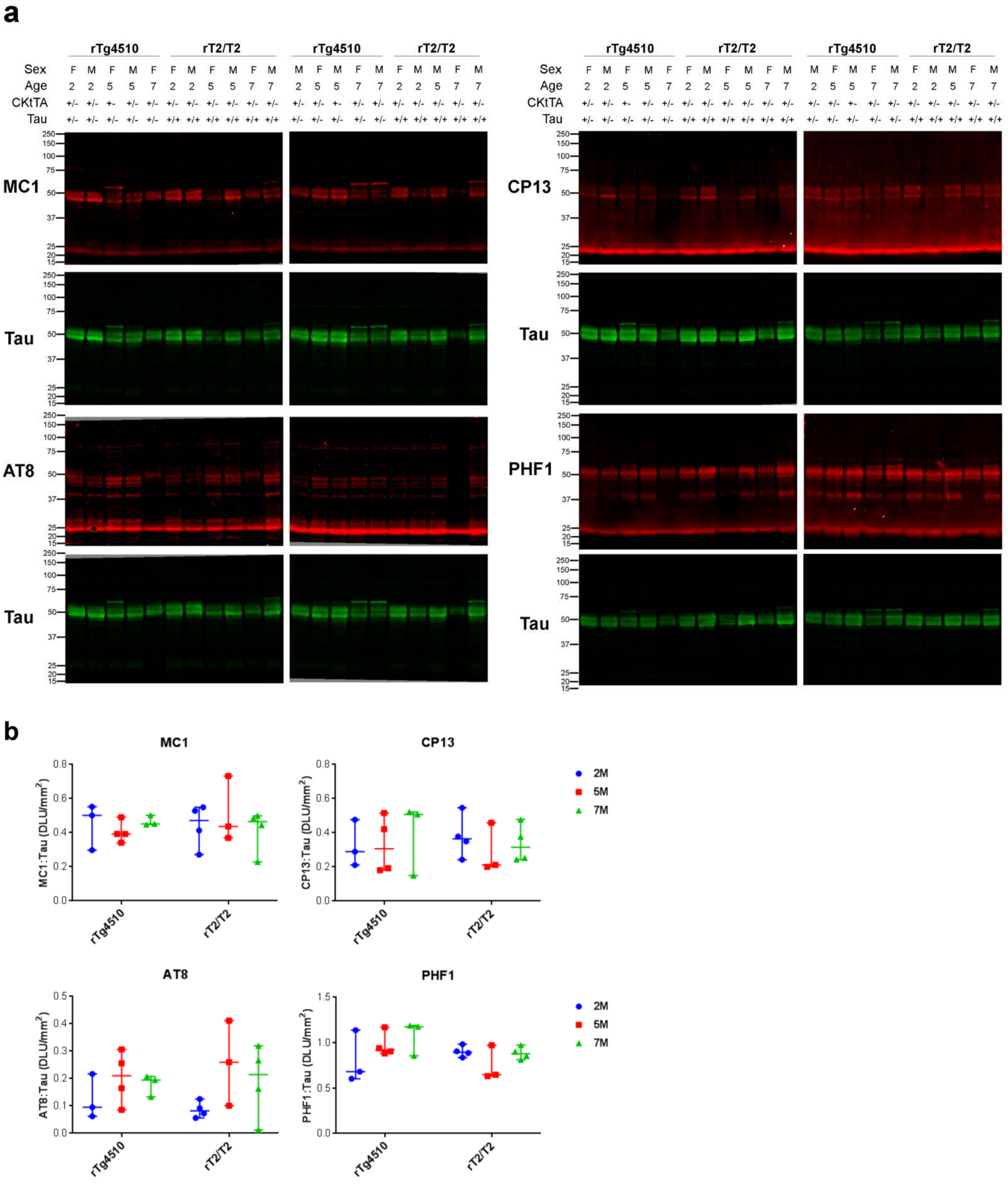

Supplementary Figure 10. Quantification of pathological Tau in rTg4510 and rT2/T2 forebrains relative to human tau. Western blots (a) and densitometric quantification (b) of pathological Tau relative to human Tau (ab74391) levels in rTg4510 (*n* = 3 2M, 4 5M, 4 7M) and rT2/T2 (*n* = 4 2M, 3 5M, 4 7M) mice. (a) Antibodies for pathological Tau included MC1 (Tau aa 7-9 and 326-330), AT8 (Tau pSer^{202}/pThr^{205}), CP13 (Tau pSer^{202}), and

PHF1(Tau pSer^{396}/pSer^{404}). Human tau specific (ab74391, Abcam) immunoblots used for normalization are shown below the appropriate pathological Tau immunoblot. The AT8 antibody gave a weak signal. (b) Two-Way ANOVAs were conducted on MC1:Tau, AT8:Tau, CP13:Tau, and PHF1:Tau signal at 2, 5, and 7 months of age, and revealed no main effects of age or transgene for any measure. Graphs display group medians ± 95% confidence intervals. Source data are provided as a Source Data file.

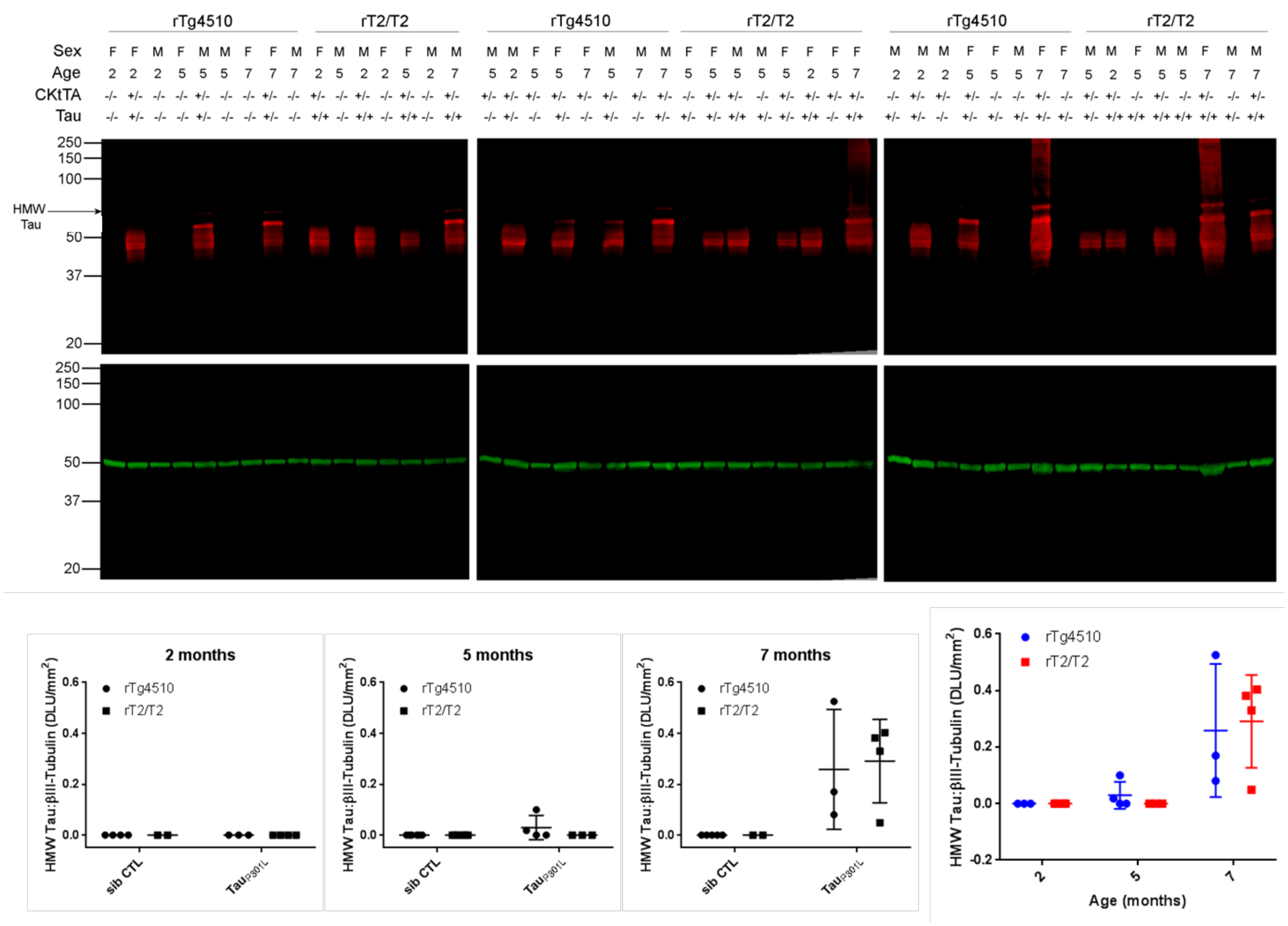

Supplementary Figure 11. High-molecular-weight (HMW) human tau in RIPA-insoluble, sarkosyl-insoluble fraction increases with age in both rTg4510 and rT2/T2. Protein from rTg4510 (*n* = 3 2M, 4 5M, 4 7M), rT2/T2 (*n* = 4 2M, 3 5M, 4 7M), and sibling control mouse brains was extracted in RIPA buffer. RIPA-insoluble protein was solubilized in 1% sarkosyl, and the remaining pellet was kept as the RIPA-insoluble, sarkosyl-insoluble fraction. Top: Western blots showing tau (Tau13, BioLegend) and βIII-tubulin in the RIPA-insoluble, sarkosyl-insoluble fraction. High-molecular-weight (HMW) tau consists of one band above 50 kDa. Genotypes, age, and sex are shown for each sample. Bottom: The HMW tau band was quantified relative to βIII-tubulin by densitometry for sibling controls (sib CTL) and Tau_{P301L} –expressing mice. Hemizygous rT2 samples were excluded from all analyses. Mann-Whitney tests comparing rTg4510 to rT2/T2 showed no significant differences at any age. Source Data are provided as a Source Data file.

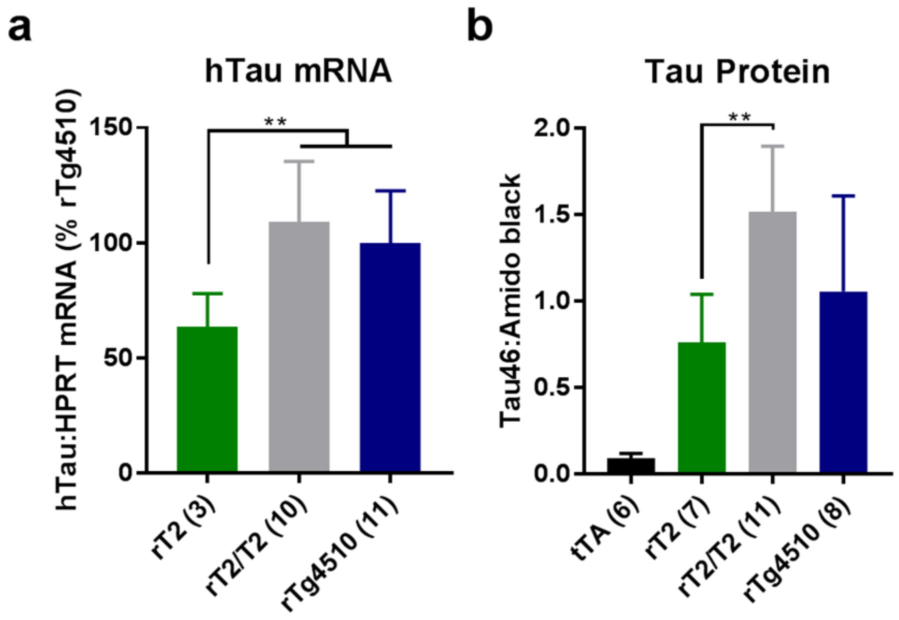

Supplementary Figure 12. Human tau expression levels in tau-hemizygous rT2 forebrains compared to rT2/T2 and rTg4510. (a) Relative qRT-PCR experiments on tau mRNA from mouse forebrains show that rT2 express 63% of rTg4510 and 58% of rT2/T2 levels. A One-way ANOVA revealed significantly lower levels in rT2 than the other two lines (F (2, 27) = 7.984, p=0.0019). Samples were run in duplicate and the experiment was replicated. (b) Protein from mouse forebrains was treated with phosphatase and analyzed by semi-quantitative western blot relative to total protein as a more accurate alternative to single-protein loading controls[1]. A pan-tau antibody (Tau46) was used to quantify fold-overexpression of human tau relative to endogenous tau in tTA mice, and was found to be 8.5-fold in rT2, 17-fold in rT2/T2, and 12-fold in rTg4510. Note that the fold-overexpression is twice as high in rT2/T2 as rT2, as would be predicted by the hemizygosity of the rT2 line. A One-way ANOVA excluding tTA mice revealed a significant difference between rT2 and rT2/T2 (F (2, 23) = 7.378, p=0.0033). Data represent mean ± SD. *N* for each group in parentheses. **p<0.01 Source data are provided as a Source Data file.

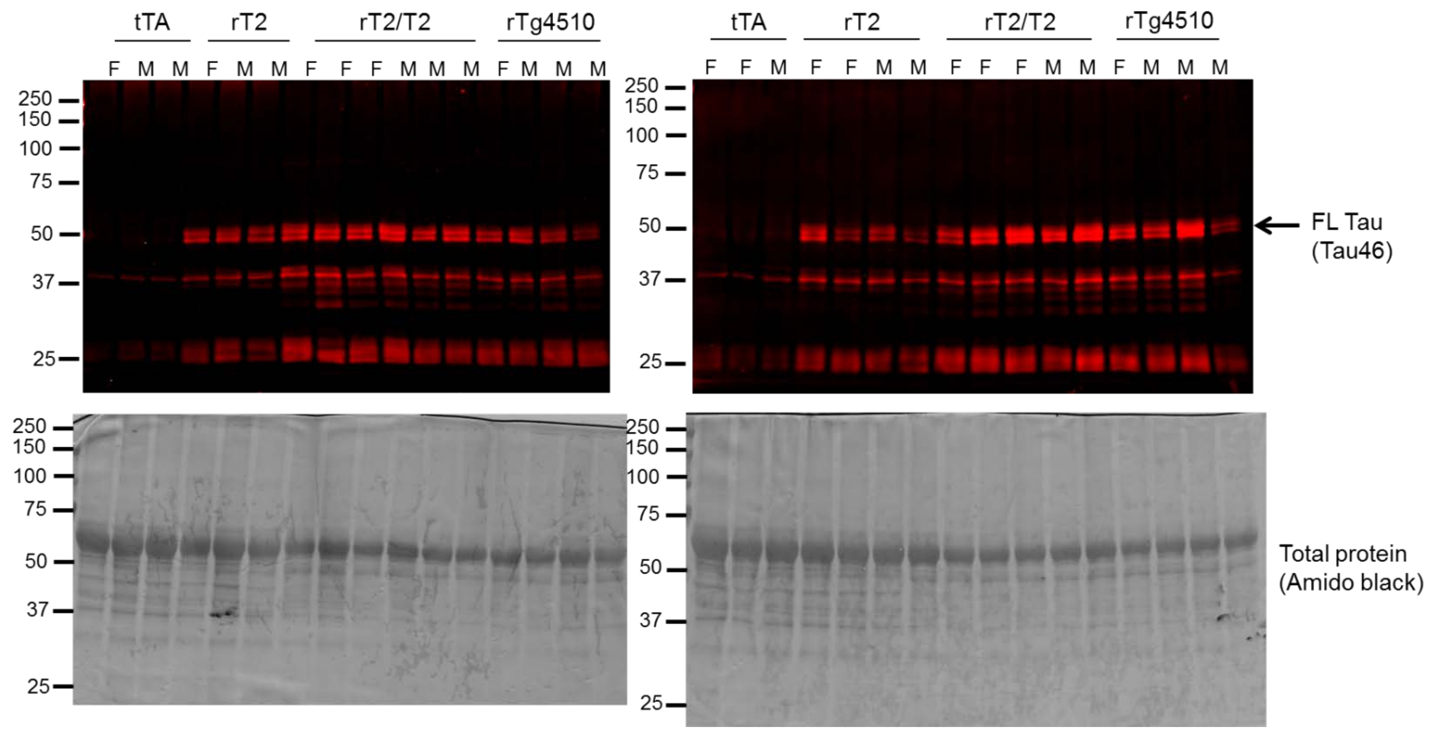

Supplementary Figure 13. Western blot images for Figure 1b. Top blots show human and mouse tau detected with Tau46 in phosphatase-treated samples from tTA, tau-hemizygous rT2, tau-homozygous rT2/T2, and rTg4510 forebrains. Amido black total protein stain was used as a more accurate alternative to high-abundance single protein loading controls[1] and is shown on the bottom. Only full-length tau (50 kDa) was quantified, while lower bands may represent tau cleavage products and/or non-specific signal. The multiple tau bands around 50 kDa likely indicate the presence of other post-translationally modified forms of tau besides phosphorylated tau. Bands 37 kDa or lower are likely tau fragments or degraded tau. Densitometric quantification is shown in Figure 1b and Supplementary Figure 12b.

TABLES

Supplementary Table 1. Primer sequences used for relative RT-qPCR.

**Target**	**Primers**	
	Forward	Reverse
Tau	5'- CTACACCATGCACCAAGACC -3'	5'- TGCTTTTACTGACCATGCGA - 3'
FGF14 variant V1	5'- CATCTTCGGCCTCAAGAAGC	5'- TCAGAACACCTGAGGATCTGGC -

	-3’	3’
FGF14 variant V2	5’- GGCCTCTTCTTTCTCAGGGT -3’	5’- CATGCAAGGATGGTTCTCGG -3’
FGF14 variant X1	5’- TGCTAGGCTGAGCAATGTAG -3’	5’- CCTTGGATCTTCGATAGGGC -3’
FGF14 variant X2	5’- ATGCTGCAGTGTCTTTGTGG -3’	5’- TCACAGGGGATGCTCAGAAG -3’
HPRT	5’- GCTGGTGAAAAGGACCTCT -3’	5’- CCACAGGACTAGAACACCTGCTA -3’

Supplementary Table 2. PCR cycling conditions using for relative RT-qPCR.

**Tau**			**FGF14 variant V1**			**FGF14 variant V2**			**FGF14 variant X1**			**FGF14 variant X2**		
#	°C	Time	#	°C	Time	#	°C	Time	#	°C	Time	#	°C	Time
1	95	10:00	1	95	10:00	1	95	10:00	1	95	10:00	1	95	10:00
32	95	00:10	45	95	00:10	40	95	00:10	40	95	00:10	45	95	00:10
	55	00:10		56	00:15		52	00:15		57	00:15		56	00:15
	72	00:10		72	00:25		72	00:28		72	00:12		72	00:16

(#) number of cycles, (°C) temperature, Time (minutes:seconds)

Supplementary Table 3. Primer sequences used to amplify Tau transgene junctions.

**Junction Product**	**Primers**		**Nested primers**	
	**Forward**	**Reverse**	**Forward**	**Reverse**
5’	5’- CAGCATCAAGCCCCAGAGAA -3’	5’- ACGCTATCTGTGCAAGGTCC -3’	5’- GCTCTGGATTTCTGTGAGCCT -3’	5’- GTAACCGGCCTCTTCATCGG -3’
3’	5’- TCTGGAACTCACCAGGTAATC -3’	5’- AACAAGGGTGGTTTTCAAGAG -3’	N/A	N/A
Tail-Head	5’- TCTGGAACTCACCAGGTAATC -3’	5’- CATGGGTAGGCTCACACAGT -3’	5’- ACCACGCTATCTGTGCAAG -3’	5’- GGATGACTGCATCTGTCAAG -3’
Head-Head	5’- GAGCGAGGAAGCCATCGTT -3’	5’- CAGATAGCGTGGTCCGGC -3’	N/A	N/A

Supplementary Table 4. PCR cycling conditions used for non-nested PCR reactions for Tau transgene junctions.

**5'**			**3'**			**Tail-Head**			**Head-Head**		
#	°C	Time	#	°C	Time	#	°C	Time	#	°C	Time
1	94	08:00	1	95	02:00	1	94	08:00	1	95	02:00
33	94	01:15	35	95	00:20	33	95	00:20	35	95	00:20
	55	00:30		53.6	00:20		56	00:20		54	00:20
	72	01:00		72	00:30		72	00:36		72	00:30
1	72	03:00	1	72	03:00	1	72	03:00	1	72	03:00

(#) number of cycles, (°C) temperature, Time (minutes:seconds)

Supplementary Table 5. PCR cycling conditions used for nested PCR reactions for Tau transgene junctions.

**5' Nested**			**Tail-Head Nested**		
#	°C	Time	#	°C	Time
1	94	08:00	1	94	08:00
33	94	01:15	35	95	00:20
	55	00:30		57.2	00:20
	72	01:00		72	00:30
1	72	03:00	1	72	03:00
1	4	∞	1	4	∞

(#) number of cycles, (°C) temperature, Time (minutes:seconds)

Supplementary Table 6. Primer sequences used to amplify CaMKIIα-tTA transgene junctions.

Junction	Forward	Reverse
Vector-TgHead	5' – AAGCCCTCCCGTATCGTAGTTAT – 3'	5' – GATGAAGACTTGACCTTGCCTC – 3'
TgHead-Vector	5' – TCCACTAGGCTCCCAAGTCAT – 3'	5' – AAGGCGATTAAGTTGGGTAACG – 3'
TgTail-TgHead	5' – AGGAACCTTACTTCTGTGGTGTG – 3'	5' – TCCACTAGGCTCCCAAGTCAT – 3'
TgTail-*Ptprn2*	5' – AGGAACCTTACTTCTGTGGTGTG – 3'	5' – CCCATTTACAGGGAAAATAAAGCC – 3'
Vipr2-TgTail	5' – CTCAGTGGGACAAGCTACCAAA –	5' – AAATAGCTTCTGCCGAGAGTCC – 3'

	3’	

Supplementary Table 7. PCR cycling conditions used for CaMKIIα-tTA transgene junctions.

**Vector-TgHead**			**TgHead-Vector**			**TgTail-TgHead**			**TgTail-*Ptprn2***			***Vipr2*-TgTail**		
#	°C	Time	#	°C	Time	#	°C	Time	#	°C	Time	#	°C	Time
1	95	02:00	1	95	02:00	1	95	02:00	1	95	02:00	1	95	02:00
30	95	00:20	30	95	00:20	30	95	00:20	30	95	00:20	30	95	00:20
	52	00:30		52	00:20		52	00:20		52	00:20		52	00:20
	72	00:45		72	00:30		72	00:50		72	00:43		72	00:30
1	72	03:00	1	72	03:00	1	72	03:00	1	72	03:00	1	72	03:00

(#) number of cycles, (°C) temperature, Time (minutes:seconds)

Supplementary References

1. Aldridge, G.M., Podrebarac, D.M., Greenough, W.T., & Weiler, I.J., The use of total protein stains as loading controls: an alternative to high-abundance single-protein controls in semi-quantitative immunoblotting. *J Neurosci Methods* 172 (2), 250-254 (2008).
