# Supplementary material for: Factors other than hTau overexpression that contribute to tauopathy-like phenotype in rTg4510 mice
Source: Nat Commun. 2019 Jun 6;10:2479. doi: 10.1038/s41467-019-10428-1 (PMC6554306; doi:10.1038/s41467-019-10428-1)
Supplement: Supplementary file 2 — Reporting Summary [file 41467_2019_10428_MOESM2_ESM.pdf]

nature research

Corresponding author(s): Michael Koob

Last updated by author(s): Apr 3, 2019

Reporting Summary

Nature Research wishes to improve the reproducibility of the work that we publish. This form provides structure for consistency and transparency in reporting. For further information on Nature Research policies, see Authors & Referees and the Editorial Policy Checklist.

Statistics

For all statistical analyses, confirm that the following items are present in the figure legend, table legend, main text, or Methods section.

n/a	Confirmed	
☐	☒	The exact sample size (n) for each experimental group/condition, given as a discrete number and unit of measurement
☐	☒	A statement on whether measurements were taken from distinct samples or whether the same sample was measured repeatedly
☐	☒	The statistical test(s) used AND whether they are one- or two-sided<br>*Only common tests should be described solely by name; describe more complex techniques in the Methods section.*
☐	☒	A description of all covariates tested
☐	☒	A description of any assumptions or corrections, such as tests of normality and adjustment for multiple comparisons
☐	☒	A full description of the statistical parameters including central tendency (e.g. means) or other basic estimates (e.g. regression coefficient) AND variation (e.g. standard deviation) or associated estimates of uncertainty (e.g. confidence intervals)
☐	☒	For null hypothesis testing, the test statistic (e.g. F, t, r) with confidence intervals, effect sizes, degrees of freedom and P value noted<br>*Give P values as exact values whenever suitable.*
☒	☐	For Bayesian analysis, information on the choice of priors and Markov chain Monte Carlo settings
☒	☐	For hierarchical and complex designs, identification of the appropriate level for tests and full reporting of outcomes
☒	☐	Estimates of effect sizes (e.g. Cohen's d, Pearson's r), indicating how they were calculated

Our web collection on statistics for biologists contains articles on many of the points above.

Software and code

Policy information about availability of computer code

Data collection	LightCycler® 480 Software release 1.5.0 SP3 was used for RT-qPCR data collection. Western blot fluorescent images were captured using Image Studio Software (Odyssey) and quantified using OptiQuant version 3 software. IHC images were capture using PixeLINK Capture SE software version 2.2 (Firewire camera release 4, Copyright © 2000-2006). All sequence analyses were conducted using the University of Minnesota's installation of the Galaxy web-based suite of software, including the BLAT alignment tool Bowtie2. The Integrative Genomic Viewer tool and SPADES were used to further analyze sequences. For FISH image capture, FISHView ASI software was used. For Open Field behavioral testing, Noldus Ethovision XT 10.0 (Noldus Information Technology) was used to track animals.
Data analysis	Graphpad Prism version 6.00 and R programming language were used for statistical tests and LightCycler® 480 Software release 1.5.0 SP3 was used for RT-qPCR data analysis.

For manuscripts utilizing custom algorithms or software that are central to the research but not yet described in published literature, software must be made available to editors/reviewers. We strongly encourage code deposition in a community repository (e.g. GitHub). See the Nature Research guidelines for submitting code & software for further information.

Data

Policy information about availability of data

All manuscripts must include a data availability statement. This statement should provide the following information, where applicable:

- Accession codes, unique identifiers, or web links for publicly available datasets
- A list of figures that have associated raw data
- A description of any restrictions on data availability

Sequence data that support the findings of this study (e.g., Fig. 3 and 5, and Supplementary Fig. 2 and 3) have been deposited in GenBank with the primary accession codes MF989990 (Tg4510 transgene) [https://www.ncbi.nlm.nih.gov/nuccore/MF989990], MF989991 (head-to-tail transgene junction) [https://www.ncbi.nlm.nih.gov/nuccore/MF989991], MF989992 (head-to-head transgene junction) [https://www.ncbi.nlm.nih.gov/nuccore/MF989992], MF989993 (transgene array - Fgf14 promoter region junction) [https://www.ncbi.nlm.nih.gov/nuccore/MF989993], and MF989994 (transgene array – Fgf14 intron junction) [https://www.ncbi.nlm.nih.gov/nuccore/MF989994]. Accession numbers for tTA-TgINDEL sequences are MK674482 (CamKII-tTA transgene_monomer), MK674483

(CamKII-tTA Tg-Vector-Tg junction), MK674484 (CamKII-tTA_Tg-Ptrn2_junction), MK674485 (Vipr2-CamKII-tTA Tg junction), MK674486 (CamKII-tTA Tg head-to-tail junction Type 1), MK674487 (CamKII-tTA Tg head-to-tail junction Type 2), MK674488 (CamKII-tTA Tg head-to-tail junction Type 3).
The source data underlying Figs 1a-d, 2a-b, 4a-e, 6b, and 7a-e and Supplementary Figs 9b, 10b, 11 and 12a-b are provided as a Source Data file, which can be accessed at https://doid.gin.g-node.org/3730d0c4d63bcb133623ce038568c7c0/ (DOI: 10.12751/g-node.3730d0).

Field-specific reporting

Please select the one below that is the best fit for your research. If you are not sure, read the appropriate sections before making your selection.

☒ Life sciences ☐ Behavioural & social sciences ☐ Ecological, evolutionary & environmental sciences

For a reference copy of the document with all sections, see nature.com/documents/nr-reporting-summary-flat.pdf

Life sciences study design

All studies must disclose on these points even when the disclosure is negative.

Sample size	The samples sizes used were estimated based on prior studies using the rTg4510 mouse, which the lab created. For the novel rT2/T2 line, sample sizes similar to or greater than rTg4510 were used.
Data exclusions	hTau hemizygous rT2/T2 littermates were excluded from western blot densitometry analyses for Supplementary Fig. 9 and Supplementary Fig. 11 because there was not a large enough n to include this group in statistical analyses.
Replication	All attempts at replication were successful.
Randomization	Samples were added to RT-qPCR plates in random order. Otherwise, randomization was not relevant to the present study because experimental groups were determined by genotypes of the animals.
Blinding	The experimenter was blinded to sample identity for scoring of tau histopathology severity for IHC semi-quantification, for nest scoring in nest-building behavioral testing, and during open field behavioral testing. For other experiments, investigators were not blinded.

Reporting for specific materials, systems and methods

We require information from authors about some types of materials, experimental systems and methods used in many studies. Here, indicate whether each material, system or method listed is relevant to your study. If you are not sure if a list item applies to your research, read the appropriate section before selecting a response.

Materials & experimental systems

n/a	Involved in the study	
☐	☒	Antibodies
☒	☐	Eukaryotic cell lines
☒	☐	Palaeontology
☐	☒	Animals and other organisms
☒	☐	Human research participants
☒	☐	Clinical data

Methods

n/a	Involved in the study	
☒	☐	ChIP-seq
☒	☐	Flow cytometry
☒	☐	MRI-based neuroimaging

Antibodies

Antibodies used	For immunoblotting, the following primary antibodies were used: Tau13 (BioLegend, Cat# MMS-520R, Lot# B205140), GAPDH (14C10) (Cell Signaling Technology, Cat# 2118S, Lot# 10), beta-III-tubulin (ProSci #79-720) and GAPDH(GA1R) (Thermo Scientific #MA5-15738).<br><br>For immunohistochemistry and immunoblotting, the following primary antibodies were obtained from Dr. P Davies: CP13, MC1, PHF1. An AT8 antibody was also used (ThermoFisher, Cat# MN1020).<br><br>The secondary antibodies used for immunoblotting were IRDye-linked Goat anti-mouse 800CW (LI-COR Biosciences, Cat# 925-32210, Lot# C40826-04) and Goat anti-rabbit 680LT (LI-COR Biosciences, Cat# 926-68021, Lot# C40916-01).<br><br>The secondary antibody used for immunohistochemistry was biotinylated anti-mouse (Vector Laboratories, Burligame, CA).
Validation	Details on the validation of Tau13 are in the AlzForum online antibody database, http://www.alzforum.org/antibodies/tau-13-tau-13-0. The GAPDH and beta-III-tubulin antibodies have been validated for western blotting using various cell lines and mouse brain homogenate.<br><br>Details on the validation of all of these antibodies are in the AlzForum online antibody database: CP13 (http://www.alzforum.org/antibodies/tau-phos-ser202-cp13), MC1 (http://www.alzforum.org/antibodies/tau-mc1), PHF1 (http://

www.alzforum.org/antibodies/tau-phos-ser396ser404-phf-1).

AT8 has been validated for western blotting and used, for example, in the following study: van Abel et al., 2012, Neuroscience Letters.

Animals and other organisms

Policy information about studies involving animals; ARRIVE guidelines recommended for reporting animal research

Laboratory animals	The animals used for this study were all Mus musculus. The rT2/T2 and rTg4510 lines were mixed FVB/NJ and 129S6 strains. The Tg4510 mice were FVB/NJ. Non-transgenic mice were either mixed FVB/NJ-129S6 or FVB/NJ. Both males and females were used for these studies. Mice of the following ages were used: 8 weeks, 2 months, 5 months, and 7 months.
Wild animals	The study did not involve wild animals.
Field-collected samples	The study did not involve samples collected from the field.
Ethics oversight	All experiments with animals described in this study were approved by and conducted in full accordance with the American Association for the Accreditation of Laboratory Animal Care and the Institutional Animal Care and Use Committee at the University of Minnesota.

Note that full information on the approval of the study protocol must also be provided in the manuscript.
